# Rethinking solitude: 5 decades of data reveal social tolerance in a traditionally solitary felid

**DOI:** 10.1093/jmammal/gyag058

**Published:** 2026-07-07

**Authors:** Aidan B Branney, Thomas J Yamashita, Lisanne S Petracca, Jason V Lombardi, Daniel G Scognamillo, Terry L Blankenship, Arturo Caso, Clay V Fischer, Lon I Grassman, Alexandria Hiott, Jennifer M Korn, Linda L Laack, John P Leonard, Victoria L Locke, Ashley Reeves, Eric L Rulison, Landon R Schofield, Maksim Sergeyev, Zachary M Wardle, Justin P Wied, Michael E Tewes

**Affiliations:** Caesar Kleberg Wildlife Research Institute, Texas A&M University-Kingsville, 700 University Boulevard, MSC 218, Kingsville, TX 78363, United States; Warnell School of Forestry and Natural Resources, University of Georgia, 180 East Green Street, Athens, GA 30605, United States; Caesar Kleberg Wildlife Research Institute, Texas A&M University-Kingsville, 700 University Boulevard, MSC 218, Kingsville, TX 78363, United States; Department of Fish, Wildlife, and Conservation Biology, Colorado State University, 901 Amy Van Dyken Way, Fort Collins, CO 80523, United States; Rocky Mountain Research Station, United States Forest Service, 240 West Prospect Street, Fort Collins, CO 80526, United States; Caesar Kleberg Wildlife Research Institute, Texas A&M University-Kingsville, 700 University Boulevard, MSC 218, Kingsville, TX 78363, United States; Caesar Kleberg Wildlife Research Institute, Texas A&M University-Kingsville, 700 University Boulevard, MSC 218, Kingsville, TX 78363, United States; California Department of Fish and Wildlife, Wildlife Health Laboratory, 1701 Nimbus Road, Rancho Cardova, CA 95670, United States; Caesar Kleberg Wildlife Research Institute, Texas A&M University-Kingsville, 700 University Boulevard, MSC 218, Kingsville, TX 78363, United States; Natural Resources Institute, Texas A&M University, 1001 Holleman Dr E, College Station, TX 77840, United States; Safari Club International Foundation, 654 Richland Hills, Suite 160, San Antonio, TX 78245, United States; Caesar Kleberg Wildlife Research Institute, Texas A&M University-Kingsville, 700 University Boulevard, MSC 218, Kingsville, TX 78363, United States; Welder Wildlife Refuge, 10429 Welder Wildlife, HWY 77, Sinton, TX 78387, United States; Caesar Kleberg Wildlife Research Institute, Texas A&M University-Kingsville, 700 University Boulevard, MSC 218, Kingsville, TX 78363, United States; Predator Conservation AC, Calzada al desierto de los leones 4448, Mexico City, D.F 0100, Mexico; Caesar Kleberg Wildlife Research Institute, Texas A&M University-Kingsville, 700 University Boulevard, MSC 218, Kingsville, TX 78363, United States; ICF 5 Lakeway Ct, #200, Lakeway, TX 78734, United States; Caesar Kleberg Wildlife Research Institute, Texas A&M University-Kingsville, 700 University Boulevard, MSC 218, Kingsville, TX 78363, United States; ICF, 823 Congress Avenue, Suite 1010, Austin, TX 78701, United States; Caesar Kleberg Wildlife Research Institute, Texas A&M University-Kingsville, 700 University Boulevard, MSC 218, Kingsville, TX 78363, United States; Caesar Kleberg Wildlife Research Institute, Texas A&M University-Kingsville, 700 University Boulevard, MSC 218, Kingsville, TX 78363, United States; Johnson Engineering, 2122 Johnson Street, Fort Meyers, FL 33901, United States; Caesar Kleberg Wildlife Research Institute, Texas A&M University-Kingsville, 700 University Boulevard, MSC 218, Kingsville, TX 78363, United States; U.S. Fish and Wildlife Service, 22817 Ocelot Road, Los Fresnos, TX 78566, United States; Travis County Natural Resources & Environmental Quality, PO Box 1748, Austin, TX 78701, United States; Caesar Kleberg Wildlife Research Institute, Texas A&M University-Kingsville, 700 University Boulevard, MSC 218, Kingsville, TX 78363, United States; Oak Street Health, Flagstaff, AZ, United States; Caesar Kleberg Wildlife Research Institute, Texas A&M University-Kingsville, 700 University Boulevard, MSC 218, Kingsville, TX 78363, United States; East Foundation, 200 Concord Plaza Dr., San Antonio, TX 78216, United States; Caesar Kleberg Wildlife Research Institute, Texas A&M University-Kingsville, 700 University Boulevard, MSC 218, Kingsville, TX 78363, United States; California Department of Transportation, 1120 N Street, Sacramento, CA 95814, United States; East Foundation, 200 Concord Plaza Dr., San Antonio, TX 78216, United States; Caesar Kleberg Wildlife Research Institute, Texas A&M University-Kingsville, 700 University Boulevard, MSC 218, Kingsville, TX 78363, United States; Rocky Mountain Research Station, United States Forest Service, 240 West Prospect Street, Fort Collins, CO 80526, United States; Warner College of Natural Resources, Colorado State University, 1401 Campus Delivery, Fort Collins, CO 80523, United States; Caesar Kleberg Wildlife Research Institute, Texas A&M University-Kingsville, 700 University Boulevard, MSC 218, Kingsville, TX 78363, United States; Division of Habitat and Species Conservation, Florida Fish and Wildlife Conservation Commission, 401 Shell Island Road, Naples, FL 34113, United States; Caesar Kleberg Wildlife Research Institute, Texas A&M University-Kingsville, 700 University Boulevard, MSC 218, Kingsville, TX 78363, United States; Department of Ecology and Conservation, Texas A&M AgriLife, Texas A&M University, 600 John Kimbrough Boulevard, College Station, TX 77843, United States; Caesar Kleberg Wildlife Research Institute, Texas A&M University-Kingsville, 700 University Boulevard, MSC 218, Kingsville, TX 78363, United States

**Keywords:** AKDE, Bobcat, felid, carnivore, home range, overlap, proximity, sociality, AKDE, área de actividad, félido, lince rojo, proximidad, sociabilidad, superposición

## Abstract

Historically, felids are thought to follow a social structure where individuals are solitary and limit interactions with other individuals of the same sex. Behaviorally it is understood that individual male cats defend territories from others, which limits social interactions between individuals outside of reproduction. However, there are frequent reports that felid populations exhibit more social tolerance than previously described. In South Texas, Bobcat (*Lynx rufus*) densities are suspected to be high and observational evidence of trapping rates and camera trap data indicate that there may be a high degree of overlap among home ranges of bobcats in South Texas. Using data from VHF and GPS collared bobcats collected between 1985 to 2024, we investigated Bobcat home range overlap and proximity across South Texas. We calculated 95% autocorrelated kernel density estimates and quantified the degree of home range overlap between conspecific individuals. For those individual pairs that had > 13.2% overlap (the mean overlap of all pairs), we estimated whether individuals moved independently, avoided each other, or moved closer to one another. From 1981 to 2024, the average Bobcat home range was 7.91 km^2^ (95% confidence interval = 6.43 to 9.62 km^2^) and did not significantly change for males or females across decades. We observed 102 instances of home range overlap, 59 in VHF-monitored individuals and 43 of them in GPS-monitored bobcats. From our proximity analysis of GPS-monitored bobcats, individuals primarily moved independently of one another but did not avoid each other as much as might be expected, especially in same-sex comparisons. Using a long-term dataset on bobcats, we reveal that bobcats are not inherently solitary within our study system. Our work provides a framework for examining social interactions in other traditionally solitary animals.

## Teaser

Using one of the largest spatial data sets for bobcats (*Lynx rufus*) ever collected (1980s to present) in South Texas, we evaluated Bobcat home range size across decades, calculated home range overlap across individual bobcats, and investigated how bobcats moved in relation to one another within those overlaps to test assumptions in sociality. Overall Bobcat home ranges within our study area have not changed significantly in size in 5 decades of monitoring. Bobcat home range overlaps were primarily male–female; however, same sex overlaps were observed more than expected. Finally, while bobcats did not spatially aggregate within their home range overlaps, they did not avoid one another, suggesting a degree of spatial tolerance within their home ranges.

## Resumen

Históricamente, se considera que los félidos siguen una estructura social en la que los individuos son solitarios y limitan las interacciones con otros individuos del mismo sexo. Desde un punto de vista conductual, se entiende que los machos defienden territorios frente a otros individuos, lo que restringe las interacciones sociales fuera del contexto reproductivo. Sin embargo, existen numerosos informes que indican que las poblaciones de félidos presentan una mayor tolerancia social de lo que se había descrito previamente. En el sur de Texas, se sospecha que las densidades de gato montés (*Lynx rufus*) son elevadas, y la evidencia observacional proveniente de tasas de captura y datos de cámaras trampa sugiere que puede existir un alto grado de solapamiento entre los ámbitos hogareños de los individuos. Utilizando datos de gatos monteses marcados con collares VHF y GPS recopilados entre 1985 y 2024, investigamos el solapamiento de ámbitos hogareños y la proximidad espacial entre individuos conspecíficos en el sur de Texas. Calculamos estimaciones de densidad kernel autocorrelacionadas al 95% (AKDE) y cuantificamos el grado de solapamiento entre ámbitos hogareños. Para aquellos pares de individuos con un solapamiento superior al 13,2% (el promedio de solapamiento entre todos los pares), estimamos si los individuos se movían de forma independiente, se evitaban o se desplazaban más cerca unos de otros. Entre 1981 y 2024, el tamaño promedio del ámbito hogareño del gato montés fue de 7.91 km^2^ (intervalo de confianza del 95% = 6.43–9.62 km^2^) y no cambió significativamente entre machos y hembras a lo largo de las décadas. Observamos 102 instancias de solapamiento de ámbitos hogareños, 59 en individuos monitoreados con VHF y 43 en gatos monteses monitoreados con GPS. A partir de nuestro análisis de proximidad en individuos con GPS, los gatos monteses se movieron principalmente de manera independiente entre sí, pero no se evitaron tanto como cabría esperar, especialmente en comparaciones entre individuos del mismo sexo. Utilizando un conjunto de datos de largo plazo sobre gatos monteses, revelamos que esta especie no es inherentemente solitaria dentro de nuestro sistema de estudio. Nuestro trabajo proporciona un marco para examinar las interacciones sociales en otros animales tradicionalmente considerados solitarios.

The degree to which an individual interacts with other individuals in the population, referred to as sociality, can have important consequences for fitness (Wolf and Weissing 2012; Brakes 2019). There are evolutionary fitness benefits to having high sociality including decreased risk of depredation (Krause and Ruxton 2002), maintaining shared food resources (Stevens and Gilby 2004), and higher reproductive output through kin selection (Hasegawa and Kutsukake 2019). Sociality can vary from short to long-term associations (Brakes 2019), with high sociality in mammals ranging across taxonomic orders including Chiroptera, Primates, Rodentia, and Artiodactyla (Hayes and Ebensperger 2016; Silk and Kappeler 2017; Rendell et al. 2019; Ripperger and Carter 2021). Of the mammalian taxonomic orders, one that is well-known for high degrees of sociality are the members of Carnivora (Finarelli and Flynn 2009).

Many families in the order Carnivora are gregarious and form complex social hierarchies (Thompson 1978; Finarelli and Flynn 2009; Bailey et al. 2013; Hunt 2022). Carnivores with high degrees of sociality can benefit in the forms of cooperative hunting, where time to acquisition of prey decreases with increased numbers searching for prey (Lamprecht 1981; Macdonald 1983; Hansen et al. 2024). Reproductive output can also be bolstered by high sociality through co-breeding and co-rearing of offspring as seen in Canidae, Hyaenidae, and Mustelidae (Leuchtenberger et al. 2014; Vullioud et al. 2019; Federico et al. 2020; SunderRaj et al. 2022). Despite these instances of high sociality amongst carnivores, Felidae historically has often been designated as solitary and antisocial in nature (Mellen 1993; Bradshaw 2016), despite more recent reports of sociality in various populations (Elbroch et al. 2017; Payne et al. 2024).

Except for lions (*Panthera leo;*  Packer et al. 1990) and cheetahs (*Acinonyx jubatus*; Durant et al. 2004), felid species were historically believed to not form social hierarchies and exhibit little degrees of sociality with other individuals not associated with reproductive success (Erofeeva and Naidenko 2012; Bradshaw 2016). Felids generally form a highly promiscuous mating system, where males defend a large home range with multiple females forming their own smaller home ranges overlapping with the home range of the male (de Azevedo and Murray 2007; Erofeeva and Naidenko 2012; Tirelli et al. 2018). Males are described as hypervigilant against rival males and will fiercely guard their territory and resources while females are believed to be not as aggressive, apart from natal defense (Bradshaw 2016; Jongman 2007). Often the threat of aggression by older males will cause young males to disperse farther than young females to establish their home ranges (de Oliveira et al. 2022). Conversely, females require high site familiarity to raise young and therefore disperse shorter distances and potentially stay near their natal home ranges (Li and Koko 2019; de Oliveira et al. 2022; Payne et al. 2024). However, despite these historical observations of low degrees of sociality, there are contradictions within Felidae and even observations of spatial and social tolerance within populations (Elbroch et al. 2017; Payne et al. 2024).

In several studies across felids, there have been instances of sociality in traditionally non-social species. Cavalcanti and Gese (2009) demonstrated that not only did male jaguars (*Panthera onca*) have home range overlap with other males but there were instances of carcass sharing among individuals. It has also been observed that older male pumas (*Puma concolor)* were tolerant of younger male pumas in their home ranges (Elbroch et al. 2016, 2017). Same sex home range overlap has also been observed in tigers (*Panthera tigris*; Liu et al. 2024), guigña (*Leopardus guigna*; Dunstone et al. 2002), and Pallas cats (*Otocolobus manul*; Ross et al. 2012). These instances of same-sex home range overlap are potentially associated with kinship (Janečka et al. 2006; Rodgers et al. 2015; Elbroch et al. 2016, 2017; Payne et al. 2024). Due to the growing and variable evidence of social tolerance among felines, we wanted examine sociality and social tolerance in a widely abundant feline in North America: the Bobcat (*Lynx rufus*).

Bobcats are the most widely distributed wild non-domestic felid in North America (Roberts and Crimmins 2010; Lavariega et al. 2022) and are classified frequently as habitat generalists as they can be found in a variety of vegetation communities and climates throughout their range (Espinosa-Flores and López-González 2017; Dunagan et al. 2019; Marrotte et al. 2020; McNitt et al. 2020). Population densities vary, but they are estimated to be particularly high in the southern extent of their range (Blankenship 2000; Heilbrun et al. 2006; Thornton and Pekins 2015). Bobcats are believed to follow traditional felid spatial patterns, where males demonstrate low proximity to other males but will exhibit home range overlap with females (Bailey 1974; Nielsen and Woolf 2001). Previous efforts to understand Bobcat social organization revealed traditional patterns of feline sociality. However, these evaluations of home range overlap and proximity are limited as they are based upon broad resolution very high frequency (VHF) data (Bailey 1974; Lawhead 1984; Nielsen and Woolf 2001; Diefenbach et al. 2006; Janečka et al. 2006). More recent investigations with GPS collars revealed patterns of same-sex overlap in space among bobcats (Melville et al. 2015; Young et al. 2019; Branney et al. 2024). With the observed patterns of substantial Bobcat home range overlap, it offers potential to evaluate the degree that bobcats exhibit sociality within calculated overlaps.

Our objective was to test the assumptions of sociality within a dense population of bobcats in the southern extent of their range using VHF and global positioning system (GPS) collar data. Specifically, we quantified the home range size of male and female bobcats across time and examined consistency in home range size through time and the degree of intersexual and intrasexual overlap between individuals. Further, for overlapping individuals, we examined the proximity of their locations to one another and assessed whether individuals are moving independently, avoiding each other, or aggregating closer to one another. We hypothesized that bobcats with overlapping home ranges would partition space and time and predicted that there would be avoidance in their proximity to one another and thus less sociality and potential interacting behavior. We leverage data across 5 decades to examine sociality and quantify space use and home range overlap for a feline considered solitary in nature.

## Methods

### Study area

**Fig. 1:**
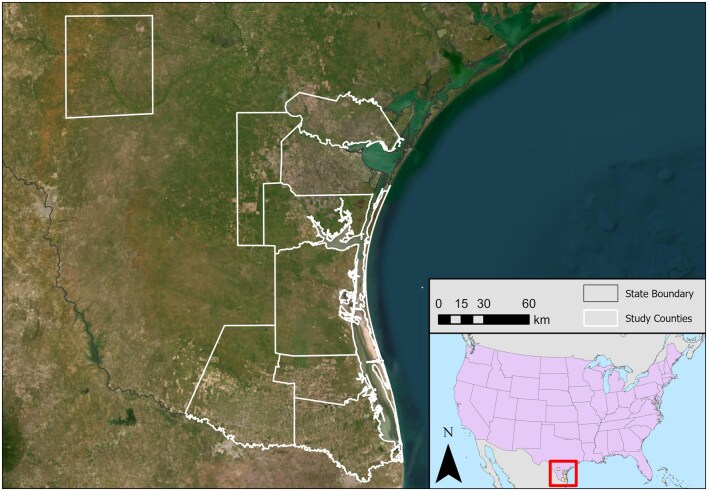
Study extent located in the rangelands of Cameron, Hildalgo, Willacy, Kenedy, Kleberg, Jim Wells, San Patricio, and La Salle counties in Texas, United States from 1985 to 2024.

Our study area consisted of 6 private ranches as well as the Welder Wildlife Refuge (WWR; private), Santa Ana National Wildlife Refuge (SANWR; federal), Lower Rio Grande Valley National Wildlife Refuge (LRGVNWR; federal), and Laguna Atascosa National Wildlife Refuge (LANWR; federal) located in Cameron, Hildalgo, Jim Wells, Kenedy, Kleberg, La Salle, San Patricio, and Willacy counties in South Texas (Fig. 1). The study region comprises the South Texas Plains and Gulf Prairies and Marshes ecoregions and represents a climate gradient from the Gulf Coast to the inland prairies and woodlands (Gould et al. 1960). The region experiences inconsistent rainfall (typically July to September) leading to episodic drought; average annual rainfall along the coast is 68 cm (Sergeyev et al. 2023) while the long-term average in La Salle County was 56 cm (Olsen et al. 2018). The climate overall is considered subtropical and semi-arid, with average temperatures ranging from 10 (January) to 38 °C (July; Norwine and Kuruvilla 2007; Olsen et al. 2018).

The 6 private properties are primarily ranching operations with varying degrees of oil and natural gas extraction. These properties are also managed for the benefit of game species and species of conservation concern. Mechanical brush clearing, prescribed fire, and systematic grazing are the most common management tools for vegetation communities.

Woody plant species found within southern Texas include Crucifixion Thorn (*Castela emoryi*), Lotebush (*Ziziphus obtusifola*), White Brush (*Aloysia gratissima*), Desert Olive (*Forestiera angustifolia*), Crucita (*Chromolaena odorata)*, Honey Mesquite (*Neltuma glandulosa*), Lime Prickly Ash (*Zanthoxylum fagara*), Live Oak (*Quercus virginiana*), Spiny Hackberry (*Celtis pallida*), Snake-eyes (*Phaulothamnus spinescens*), and Huisache (*Acacia farnesiana*; Leonard et al. 2020; Lombardi et al. 2021). Herbaceous plants include Creeping Bundleflower (*Desmanthus virgatus*), Bristlegrasses (*Setaria* spp.), Wild Petunia (*Ruellia* spp.), Gramas (*Bouteloua* spp.), and Purple Threeawn (*Aristida purpurea*; Olsen et al. 2018).

### VHF and GPS collaring

We captured 137 bobcats from 1981 to 2024 on private ranchlands, WWR, LANWR, LRGVNWR, and SANWR (87 on private lands and 50 on public land; Laack 1991; Fischer 1998; Harveson et al. 2004; Blankenship et al. 2006; Korn 2013; Leonard et al. 2020; Sergeyev et al. 2023; Branney et al. 2024). VHF collars were used from 1981–2013 and GPS collars were used from 2005 to 2024. A variety of trapping methods, chemical immobilization protocols, and collar schedules were used during this study (Supplementary Data SD1). Captured bobcats were handled under institutional animal care and use committee (IACUC) protocols and Texas Parks and Wildlife Department permits that have changed and adapted over the course of this study (Texas A&I University and Texas A&M University- Kingsville Institutional Care and Use Committee Guidelines [Protocols: IACUC 2012–12-20B-A2, 2019–2-28A-2-28B, 2023-10-20]), and Texas Parks and Wildlife Department Scientific Research permits (no. SP0190-600, SPR-1123-136).

### Analysis

From our original sample size of bobcats, we removed individuals that were monitored for l less than 30 days and had 20 points or less to effectively identify a home range and those that never established a home range during the duration of collar monitoring. We also calculated net squared displacement for each individual and visually inspected all individuals in the 75th percentile and above for possible exploratory and dispersal movements. We removed all individuals that made dispersal or exploratory movements because we were primarily interested in assessing overlap for stable, resident individuals. With our reduced sample size of 125 bobcats from 1985 to 2024, we inspected each individual and removed outlier points based on speed and turning angle. Locations that were above the 95th percentile for an individual were inspected and removed from the analysis (Calabrese et al. 2016). We also purged location data that contained horizontal dilution of precision (HDOP) values greater than 10 due to the potential inaccuracy of the location (Cargnelutti et al. 2007; Cowan et al. 2024). Approximately 15% of the GPS dataset had missing HDOP information, so all locations were assumed to be valid and accurate. After removing locations with high error, we grouped the data based on collar type (VHF or GPS), location of capture, and overlapping monitoring time. We accounted for autocorrelation structure of the data by computing semivariograms for each Bobcat in their designated group (Calabrese et al. 2016; Fleming et al. 2014). We made determinations within each grouping on whether we could pool the semivariograms based on visual assessments of similar patterns of semivariance (Fleming et al. 2014). We then created a suite of potential models for autocorrelative structure of the location data for each individual Bobcat (Fleming et al. 2014). We considered IID (bivariate normal kernel density estimator), the Ornstein-Uhlenbeck model (OU; Brownian motion restricted to finite home range), OU-F (continuous-velocity motion restricted to a finite home range), and OU-f (OU-F but with the position and velocity parameters the same). We also considered isotropic (circular) and non-isotropic (elliptical) versions of all the above models (Fleming et al. 2017). For VHF data, we only considered IID (either isotropic or non-isotropic) due to sparseness and likely lack of autocorrelation between fixes. For GPS data we considered all potential models and selected the best fitting model using corrected Akaike Information Criterion (Silva et al. 2022; Supplemental Data SD2). As our dataset ranged widely in number of locations, fix-rates, and contained issues of inconsistent sampling, we used autocorrelated kernel density estimators (AKDEs) to estimate home range size and compare home ranges among individuals and time periods (Silva et al. 2022; Tilberg and Dixon 2022; Zvidzai et al. 2022). After we determined the appropriate autocorrelation structure, we created AKDEs for each individual using the “ctmm” package in R v 4.3.1 (Fleming et al. 2014; Calabrese et al. 2016). To assess how home range size varied with decade (1980s, 1990s, 2000s, 2010s, and 2020s), sex (female and male), and collar type (VHF and GPS), we performed single analysis of variance analysis (ANOVA) on the log-transformed home ranges including all relevant 2-way interactions using Program R (R Core Team 2024). For significant effects, we calculated the group average and effect size using the meta function in “ctmm” following the method of Fleming et al. (2022). VHF estimated home ranges were created via triangulation with no inherent measure of error due to them being historical monitoring protocols.

We examined home range overlap for GPS and VHF bobcats that were monitored during the same time interval and on the same property using the overlap function in the “ctmm” package to obtain the minimum level of overlap between sympatric individuals. The overlap function calculates percent overlap based on the Bhattacharyya coefficient, which estimates the ratio of the intersection area of 2 home ranges to the average individual area to determine the percent overlap of the home ranges between 2 individuals (Winner et al. 2018; Averill-Murray et al. 2020). In addition, we were interested in computing overlap between neighboring individuals. To do this, we calculated the distance between home range centroids and identified “neighboring individuals” as those that were within the square root of the average male home range area. By calculating both overall overlap between all sympatric individuals and overlap only between neighboring individuals, we accounted for home range shape, which can be driven by features on the landscape (e.g., roads, fences, vegetation, etc.). For territorial and non-social animals, we expected low overlap among home ranges as the individuals are maintaining territories that exclude other individuals (Thompson 1978; Jakub et al. 2024). Home range overlap in carnivores has not been widely studied, so we used the mean overlap to determine the threshold for higher overlap among individuals. For bobcats, in particular, there has been no quantification of what represents low or high levels of home range overlap, so our calculations represent the first robust home range overlap calculations across time.

We conducted proximity analyses on those individuals with higher than average overlaps to evaluate Bobcat movements in relation to one another. This analysis was only conducted on GPS-collared bobcats because the coarse temporal resolution of VHF data does not allow accurate identification of locations at given points in time. Using the proximity function in the “ctmm” package, we calculated the distance between a pair of individual locations at overlapping times then fit an autocorrelation function to the distances and compared the mean-square distance between individuals to the expected distance if the 2 individuals were moving independently (Fleming et al. 2014; Calabrese et al. 2016). This method allows for accounting of variability in location data in individuals resulting in variable confidence limits. If territoriality existed, we would expect that individuals would be found further than expected for random movement whereas if individuals were exhibiting sociality, they would be closer than expected (Chhen et al. 2024).

## Results

We monitored adult bobcats on average for 313.14 d ± 293.0 (SD) across decades: (VHF= 467.98 d ± 364 (range 128 to 1,702 d); GPS= 182.12 d ± 96.9 (range 30—379 d); Supplementary Data SD3). We created AKDEs for 121/137 monitored bobcats (50 females, 67 males, and 4 of unknown sex) from 1985 to 2024. Of the 121 bobcats, 18 individuals did exploratory movements or dispersals and were excluded from further analyses. Of the individuals analyzed, 53 were monitored via VHF (20 females, 29 males, and 4 unknown) and 50 were monitored via GPS (22 females and 28 males). By decade, we assessed home range size from 14 individuals in the 1980s (2 females, 8 males, and 4 of unknown sex), 22 individuals in the 1990s (11 females and 11 males), 6 individuals in the 2000s (2 females and 4 males), 23 individuals in the 2010s (10 females, 13 males), and 38 individuals in the 2020s (17 females and 21 males).

**Fig. 2:**
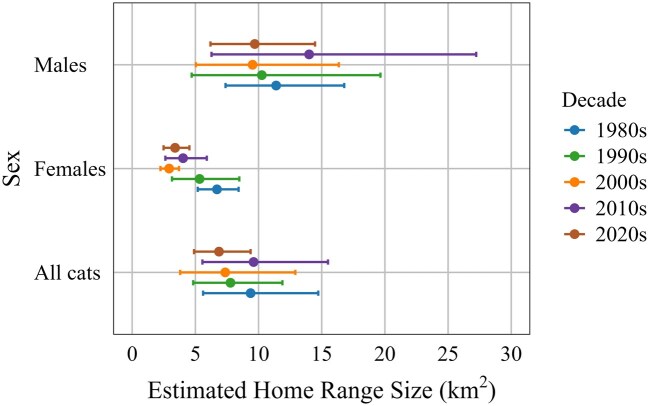
Estimates of male and female Bobcat (*Lynx rufus*) AKDE (autocorrelated kernel density estimate) home ranges partitioned by decade for 107 individuals monitored from 1985 to 2024 in South Texas.

**Fig. 3:**
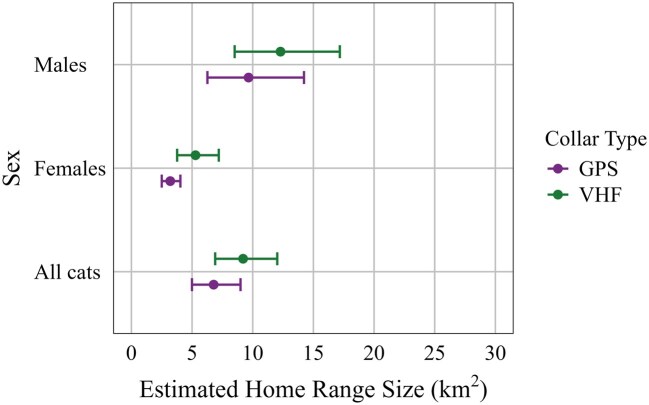
Estimates of 107 male and female Bobcat (*Lynx rufus*) AKDE (autocorrelated kernel density estimate) home ranges derived from VHF and GPS monitored individuals monitored from 1985 to 2024 in South Texas.

The average resident home range size across all individuals was 8.04 km^2^ (95% confidence interval [CI] = 6.50 to 9.82). Between sexes, male Bobcat home ranges were 2.6 (CI: 1.79 to 3.61) times larger than female home ranges (ANOVA *F*_2,104_ = 3.33, *P *= 0.04; male= 11.0 km^2^ (8.28 to 14.32), female= 4.2 km^2^ (3.37 to 5.16); Table 1). We did not identify any differences in home range size across decades (*F*_4,104_ = 0.75, *P *= 0.56; males 1980s= 11.4 km^2^ (7.39 to 16.78), 1990s = 10.3 km^2^ (4.71 to 19.65), 2000s = 9.5 km^2^ (5.04 to 16.36), 2010s = 14.0 km^2^ (6.28 to 27.23), 2020s = 9.7 km^2^ (6.20 to 14.47) and females 1980 = 6.7 km^2^ (5.19 to 8.42), 1990s = 5.3 km^2^ (3.15 to 8.48), 2000s = 2.9 km^2^ (2.24 to 3.70), 2010s = 4.0 km^2^ (2.63 to 5.91), 2020s = 3.4 km^2^ (2.48 to 4.53), Fig. 2; Supplemental Data SD3). We did not observe any differences in home range size between VHF and GPS-collared individuals (*F*_1,104_ = 0.65, *P *= 0.42; VHF= 9.2 km^2^ (6.91 to 12.02), GPS= 6.8 km^2^ (5.00 to 8.99), (Fig. 3). CIs associated with comparisons and effect sizes can be found in Supplemental Data SD4. Home range estimates did not change when we removed individuals that were monitored for longer than 1 yr.

**Table 1:** ANOVA table of differences in Bobcat (*Lynx rufus*) home range size based on sex, collar type, and decade. Home range size was determined using the “ctmm” package in R.

	SS	df	*F* value	*P* value
**Sex**	9.07	2	3.33	0.04
**Collar type**	0.88	1	0.65	0.42
**Decade**	4.08	4	0.75	0.56
**Sex × Collar type**	0.06	1	0.04	0.84
**Sex × Decade**	0.69	4	0.13	0.97
**Residual**	141.64	104		

**Fig. 4:**
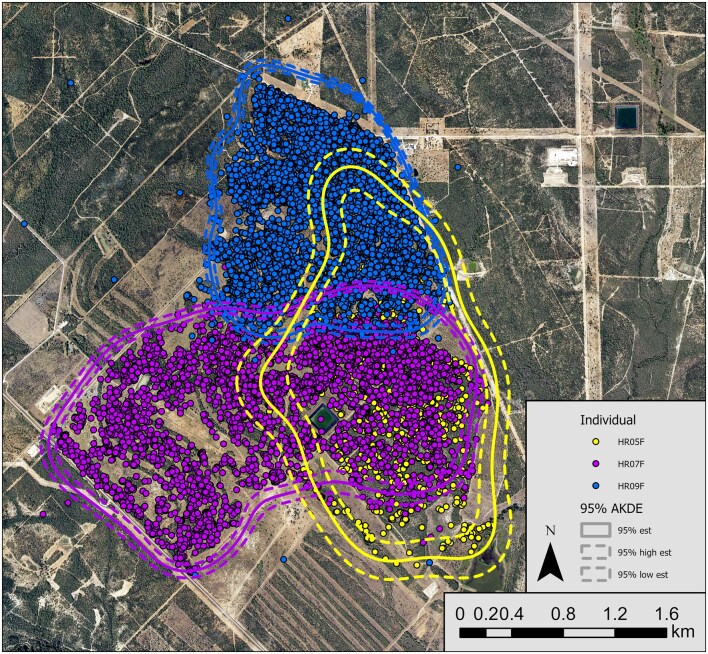
Example of same-sex AKDE home ranges and level of overlap among 3 female bobcats (HR05F, HR07F, and HR09F) monitored from May 10, 2021, to May 22, 2022, in South Texas. Overlap percentages for HR09F and HR07F were 16.1%, HR05F and HR07F 68.9%, and HR05F and HR09F 37.3%.

Of our 103 bobcats with resident home ranges, we calculated overlap for 365 pairs. When only considering neighboring individuals, 132 were between neighboring pairs. Average female–female overlap was 7.9% for GPS (*n *= 30) and 5.6% for VHF (*n *= 31). Average male–male overlap was 11.7% for GPS (*n *= 40) and 13.5% for VHF (*n *= 72). For male–female overlap, we observed 17.7% for GPS (*n *= 72) and 12.7% for VHF (*n *= 111). We observed 101 (27.7% of total) instances—87 (66% of total) were between neighboring individuals—of home range overlap that was 13.2% or greater out of 365 pairings during the duration of collar monitoring across decades (significant overlap; Fig. 4). Male–female overlap was most common with 57 occurrences (56% of all significant overlaps) across time. However, we also observed 11 instances of female–female overlap (9%), 27 instances of male–male overlap (25%), and 6 of unknown sex comparisons (6%). Of these calculated overlaps, 59 of them (58%) were calculated using VHF-derived home ranges and the remaining 43 (42%) were based on GPS-derived home ranges (Supplemental Data SD5).

**Fig. 5:**
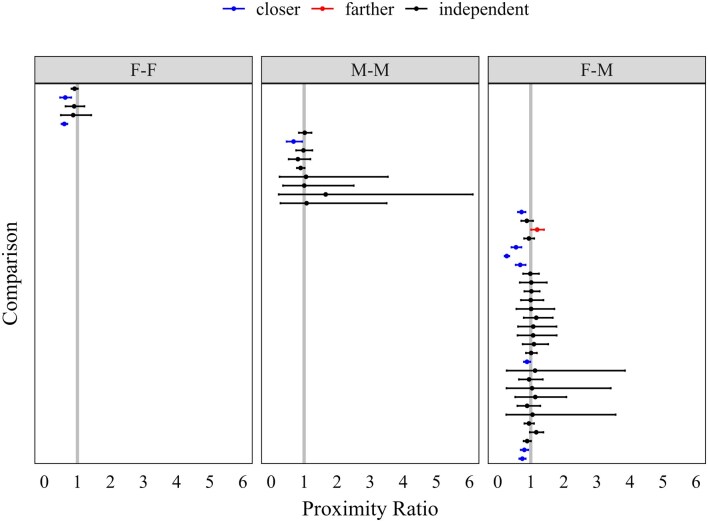
Proximity analysis of same and opposite sex overlaps observed in 43 bobcats (*Lynx rufus*) in South Texas. Values less than 1 indicate movements are closer than expected while values greater than 1 suggest avoidance. Confidence intervals that include 1 indicate independent movement.

From our proximity analysis of 43 GPS-derived home range pairings, we made comparisons of 29 male–female significant overlaps, 9 male–male significant overlaps, and 5 female–female significant overlaps. As outlined in the “ctmm” package, the proximity function generates a ratio where values less than 1 suggest spatial aggregation, values close to one represent independent movement, and values greater than 1 represent spatial avoidance. For our female–female significant overlaps, we observed 3 instances of independent movement and 2 instances of attraction (Fig. 5). For male–male significant overlaps, we observed 8 instances of independent movement and 1 instance of attraction (Fig. 5). In the male–female analyses we determined 21 instances of independent movement, 7 instances of attraction, and 1 instance of avoidance (Fig. 5).

## Discussion

Our study represents an analysis of 1 of the largest VHF and GPS datasets across time for bobcats (see Bailey 1974; Fuller et al. 1986; Litvaitis et al. 1986; Conner et al. 1992; Lovallo and Anderson 1996; Riley et al. 2003; Poessel et al. 2014; Melville et al. 2015; McNitt et al. 2020; Chamberlain et al. 2003; Plowman et al. 2006; Ferguson et al. 2009) and, importantly, suggests that bobcats possess spatial tolerance within their home ranges. The home range estimates represent the most robust for bobcats in South Texas and across its geographic range—and from our analysis has allowed us to increase our understanding of Bobcat space use and sociality in the southern extent of their distribution.

Previous average estimates for Bobcat home ranges in the southern extent of their range include 3 to 25.9 km^2^ (Lawhead 1984; Bradley and Fagre 1988; Elizalde-Arellano et al. 2012; Young et al. 2019) with most falling between 3 and 8 km^2^, which is comparable to the estimates within our study area. Surprisingly, Bobcat home range size did not change after almost 5 decades of monitoring, even though the South Texas region has experienced significant landscape changes in the same time period (Lombardi et al. 2020). There are many factors that can influence home range size, including seasonality (Morellet et al. 2013), habitat quality (Bjørneraas et al. 2012), and body mass (Lindstedt et al. 1986; Machado et al. 2017). For bobcats in our system, all these factors may play a role in their home range patterns. However, evaluating seasonality of home range size for bobcats was not an objective our study but remains an open and important question. Also, bobcats in the southern extent of their range are known to breed year-round with seasonal peaks (Sunquist and Sunquist 2002; Blankenship et al. 2006). It has also been postulated that bobcats adhere to the phenomenon of Bergmann’s rule, in which as animals occur at higher latitudes, body mass increases and resources availability can fluctuate frequently. This prediction could explain why Bobcat home range estimates closer to the poles are considerably larger (up to 108 km^2^) than those in the southern extent of their range (Wigginton and Dobson 1999; Rodríguez et al. 2008; Donovan et al. 2011). With the broad patterns we observed across Bobcat space use, we did observe differences in the space use of males and females.

Male Bobcat home ranges were larger than those of females, which is a widely observed pattern across the Bobcat distribution (Conner et al. 1992; Riley et al. 2003; Donovan et al. 2011; Melville et al. 2015; McNitt et al. 2020; Branney et al. 2024). As male felids are larger than females, they often require more resources and space to maintain a larger body size (Machado et al. 2017; Erofeeva and Naidenko 2020; Smith et al. 2025). Males also seek to maintain a home range that provides ample access to breeding opportunities with multiple female individuals (Plowman et al. 2006; Machado et al. 2017; Erofeeva and Naidenko 2020; Smith et al. 2025). Female felids, however, build their home ranges to optimize both resource requirements and survival of offspring resulting in home ranges being a fraction of the size of a male home range (Machado et al. 2017; Erofeeva and Naidenko 2020; Smith et al. 2025). In our system, these reproductive strategies across the year likely explain the size discrepancy in Bobcat space use between males and females (Smith et al. 2025). Bobcat home ranges were not different in size across decades within sexes. The rangelands of South Texas on private ranches are managed and optimized to grow prey resources (Fulbright et al. 2018). It is likely that there is a stable availability of prey for a generalist species like bobcats within this environment, meaning individuals do not have to travel far to meet their nutritional and reproductive requirements. Bobcats in South Texas may not have to maintain territories and may tolerate sharing space.

We were able to derive 102 instances of higher than average home range overlap from our sample size of bobcats that co-occurred in space and time. The majority of observed overlap was between opposite-sex individuals, as is reported in many other felids (Sliwa 2004; Dillon and Kelly 2008; Machado et al. 2017) and is likely associated with increased likelihood of reproductive success. It was unexpected to see the level of same-sex home range overlap that we observed across time, especially with 25% being male–male. Male–male (range: 35% to 39%) and female–female (range: 7% to 42%) home range overlap has been observed in bobcats within both VHF and GPS-related studies (Lawhead 1984; Conner et al. 1992; Melville et al. 2015; Young et al. 2019; Payne et al. 2024). However, to our knowledge, our study is the largest and most comprehensive analysis of Bobcat home range overlap. Our study shows similar levels of home range overlap as have previous studies, which highlights consistency in the reports of overlapping home ranges for bobcats. We observed similarity in home range estimates and overlap with both VHF and GPS derived home ranges likely because of the process used to account for autocorrelation in GPS data. During this process, data are thinned to uncorrelated locations that are distributed across an individual home range, effectively reducing the number of locations used to estimate a home range size (Fleming et al. 2014). Additionally, when using fewer points to estimate home range size from GPS data, home range estimates tend to be similar between VHF and GPS collared individuals (Pellerin et al. 2008; Kochanny et al. 2009). Therefore, we do not believe that our observations are an artifact of the technology or modeling approach used but rather an underlying ecological or genetic mechanism (Elbroch et al. 2016; Janečka et al. 2006; Payne et al. 2024). In other wild felids, kinship has manifested mixed results across taxa and sex in terms of spatial coefficients in home range overlap patterns (Nicholson et al. 2011; Rodgers et al. 2015; Schmidt et al. 2016; Elbroch et al. 2016, 2017; Payne et al. 2024). As we did not possess a comprehensive record of all genetic samples across decades, we were unable to explore potential genetic mechanisms associated with the Bobcat home range overlap in our system. However, we were able to build a foundational understanding of potential sociality through our proximity analyses across 43 pairings of GPS-monitored individuals.

In our assessment of proximity for bobcats that co-occurred in space and time, we were able to observe predominant patterns from our sample size of bobcats. For the bobcats that we were able to test, our results differed from our initial predictions on same-sex spatial and movement patterns. We expected that there would be spatial aggregation for male–female pairings and spatial avoidance for same-sex pairings (Bradshaw 2016). Independent movement was the primary pattern observed across pairings and the single avoidance pattern observed was between a male and female. Closely related female individuals are known to aggregate (Payne et al. 2024), while female–male pairs generally avoid each other outside of reproduction (Culver et al. 2010). The genetic component of male–male aggregation is not well understood in bobcats and more research is needed (Janečka et al. 2006). Kin selection is understudied in felids except lions and pumas and may be an important mechanism driving space use by other felids (Spong and Creel 2003; Elbroch et al. 2016; Chakrabarti et al. 2020). It is possible that the same-sex proximity observed in this study is due to tolerance of offspring within the home range of a parent as matrilineal assemblages have been reported in bobcats and the closely related Eurasian Lynx (*L. lynx;*  Holmala et al. 2018; Payne et al. 2024). While we fully acknowledge that our results do not support that bobcats are animals with high sociality, we did demonstrate there is more spatial tolerance for conspecifics possibly due to a genetic driver (Culver et al. 2010). We also acknowledge that bobcats in our system likely have separate core areas despite the patterns observed in home range overlap and proximity and that there is an assumption of social behavior using these measures of spatial movement (Nielsen and Woolf 2001; Young et al. 2019). However, given the small size of an area that bobcats on average occupied in our system, it is entirely possible that bobcats perceived one another more than we were able to detect. Future genetic investigations could elucidate potential drivers of why bobcats spatially aggregate and overlap within this environment.

Broad assumptions are made about the behavior of solitary mammals, especially when they are difficult to monitor. In our effort to challenge assumptions made about the solitary nature of bobcats, we generated robust home range estimates, validated what others have observed in home range overlap, and reported proximity patterns rarely discussed. Our results collectively demonstrate spatial tolerance behavior among bobcats in South Texas. From our findings, we hope that this methodology can be applicable beyond bobcats but to other traditionally solitary animals and reveal higher levels of sociality than has been typically assumed. Finally, incorporating genetic pedigrees and demographic information will allow for greater understanding of intraspecific interactions and the role sociality plays in evolutionary fitness.

## Supplementary Material

gyag058_Supplementary_Data

## Data Availability

Private land Bobcat GPS location data are not available due to confidentiality agreements with private landowners in the region. If there are questions about data access, please contact Dr. David Hewitt of the Caesar Kleberg Wildlife Research Institute.

## Supplemental data

**Supplementary Data SD1.** Description of Bobcat capture protocols.

**Supplementary Data SD2.** Model selection results of AKDE models.

**Supplementary Data SD3.** Monitoring durations of bobcats by sex, collar type, and decade.

**Supplementary Data SD4.** Population home range estimates by sex, collar type, and decade.

**Supplementary Data SD5.** Percent home range overlap for each pair of bobcats.
