# Supplementary material for: Rethinking solitude: 5 decades of data reveal social tolerance in a traditionally solitary felid
Source: J Mammal. 2026 Jul 7;107(4):751–62. doi: 10.1093/jmammal/gyag058 (PMC13416186; doi:10.1093/jmammal/gyag058)
Supplement: gyag058_Supplementary_Data [file gyag058_supplementary_data.docx]

**Supplemental Material**

Rethinking solitude: Five decades of data reveal social tolerance in a traditionally solitary felid

Branney et al.

Supplement Data SD1**:** Outline of all trapping protocols used to build the bobcat (*Lynx rufus*) VHF and GPS location data set for in South Texas from 1985 to 2024.

We captured a total of 137 adult bobcats in private ranchlands, LANWR, and SANWR (87 on private lands and 50 on public land) from 1985-2024 (Laack 1991, Fischer 1998, Harveson et al. 2004, Blankenship et al. 2006, Korn 2013, Leonard et al. 2020, Sergeyev et al. 2023, Branney et al. 2024). We captured bobcats with single-door 108 x 55 x 40 cm wire box traps (Tomahawk Trap Co., Tomahawk, WI) baited with live pigeons (*Columba livia*) or live chickens (*Gallus gallus domesticus*). Bobcats captured from 1985-1996 on LANWR and SANWR were sedated with mixtures of ketamine hydrochloride (100mg/ml; Fort Dodge Laboratories, Fort Dodge, Iowa, USA) and xylazine hydrochloride (20mg/ml; Anased, Lloyd Laboratories, Shenandoah, IA). We fitted individuals with 125g Telonics radio transmitters (Telonics Inc., Mesa, AZ) and 10-15 locations were triangulated per month during the duration of studies (Beltran and Tewes 1995, Harveson et al. 2004).

From 1993 to 2004 on the Welder Wildlife Refuge, we immobilized trapped bobcats with an intramuscular injection of 10–15 mg/kg body weight of ketamine hydrochloride and 0.05 mg/kg body weight of promazine hydrochloride (Blankenship et al. 2006). We then fit them with a 125g radio-collar (Advanced Telemetry Systems ^®^ [ATS], Isanti, Minnesota, USA). We attempted to collect > 10 locations monthly for each collared bobcat from 05:00 to 20:00.

From 2005-2017, we used intramuscular injections of Telazol (Telazol, Zoetis, Florham Park, NJ, USA) at a dosage of 5 mg per kg body weight (Shindle and Tewes 2000) or 20 mg/kg of ketamine hydrochloride (Fort Dodge Laboratories, Fort Dodge, Iowa, USA) and 0.05 mg/kg of xylazine hydrochloride (Vedco, Inc., St. Joseph, Missouri, USA; n = 5) or 20 mg/kg of ketamine hydrochloride and acepromazine maleate to sedate captured bobcats. We fitted bobcats with VHF collars manufactured by Advanced Telemetry Systems (ATS; Advanced Telemetry Systems Inc., Insanti, MN) or GPS collars manufactured by ATS, Sirtrack (Sirtrack Wireless, Dunedin, New Zealand), or Lotek (Lotek Wireless, Newmarket, Ontario, Canada). On the properties located in Willacy, Cameron, and Kennedy counties, we acquired VHF locations multiple times per month using three azimuth readings within a 30-minute period. Sirtrack collars collected locations every 11 hours while the ATS and Lotek collars were programmed according to the following schedule: one location each 24-hr period at midnight and at noon for every calendar day, with a high-frequency track period (location every 30 minutes) programmed around every full moon date and every new moon date. We programmed the ATS GPS collars with the high-frequency track schedule for a 72-hr period based on each new moon and full moon night, whereas the Lotek GPS collars were programmed with the high-frequency track schedule for a 24-hr period based on each new moon and full moon night (Leonard et al. 2020). Lotek collars in Kleberg and Jim Wells counties were programmed to record a location every 2 hours or every 4 hours (Korn 2013).

From 2019 to 2024, we immobilized bobcats with a mixture of medetomidine 10 mg/mL (target dose: 0.05-0.08 mg/kg bodyweight; Wedgewood Pharmacy: Wildlife Pharmaceuticals, Inc.; ZooPharm, Swedesboro NJ; BCP Veterinary Pharmacy, Houston TX) and ketamine hydrochloride 200 mg/mL (2.5-8 mg/kg bodyweight; Wedgewood Pharmacy: Wildlife Pharmaceuticals, Inc. and ZooPharm, Swedesboro NJ; BCP veterinary Pharmacy, Houston TX; Rockhill et al. 2011, Sergeyev et al. 2023, Branney et al. 2024). In this period within Willacy, Cameron, and Kennedy Counties, we fit bobcats with Lotek Litetrack Iridium 150g and 250g GPS-satellite collars, ATS G5-PC 310 g and G5-2A 230g collars. We recorded locations from every 30 minutes, every hour, or every two hours during the duration of the ongoing studies. For bobcats collared in La Salle County, we programmed collars to record a location every hour during the nocturnal period (19:00-7:00) and then every 2 hours during the diurnal period (7:00-19:00; Branney et al. 2024).

Supplemental Data SD2: Auto correlated kernel density estimate (AKDE) model selection for bobcat (*Lynx rufus*) home range estimation. Models are calculated by bobcat ID and includes all model structures (OUF anisotropic error, OUF error, OUf anisotropic error, OUf error, OU anisotropic error, OU error, IID, and IID anisotropic). We rank models by Akaike Information Criteria (AIC) and include Delta AIC and degrees of freedom (DOF).

| ID | Model | Delta AIC | DOF |
| --- | --- | --- | --- |
| EB20M | OUF anisotropic error | 0 | 62.5861626 |
| EB20M | OUF error | 3.62910088 | 61.3202573 |
| EB20M | OUf anisotropic error | 63.4461445 | 134.605189 |
| EB20M | OUf error | 70.5222405 | 136.997792 |
| EB20M | OU anisotropic error | 222.261834 | 34.6953023 |
| EB20M | OU error | 231.478337 | 38.505849 |
| EB22F | OUF anisotropic error | 0 | 61.9843807 |
| EB22F | OU anisotropic error | 2.65551216 | 55.6711 |
| EB22F | OU error | 139.235652 | 55.9699325 |
| EB22F | OUF error | 140.967568 | 56.902298 |
| EB22F | OUf anisotropic error | 640.999488 | 121.98923 |
| EB22F | OUf error | 706.410833 | 119.924662 |
| EB23F | OUF anisotropic error | 0 | 142.637648 |
| EB23F | OUF error | 191.001655 | 132.035444 |
| EB23F | OU anisotropic error | 577.807979 | 106.57255 |
| EB23F | OU error | 815.245701 | 191.632081 |
| EB23F | OUf anisotropic error | 1543.43981 | 641.602045 |
| EB23F | OUf error | 1780.90101 | 637.145246 |
| EB24M | OUF anisotropic error | 0 | 347.766856 |
| EB24M | OUF error | 50.3375686 | 350.112819 |
| EB24M | OU anisotropic error | 624.006335 | 242.922549 |
| EB24M | OU error | 702.312189 | 240.410875 |
| EB24M | OUf anisotropic error | 943.524761 | 926.274984 |
| EB24M | OUf error | 1010.22633 | 989.046887 |
| EB25M | OUF anisotropic error | 0 | 345.324988 |
| EB25M | OUF error | 38.6533516 | 352.6744 |
| EB25M | OU anisotropic error | 793.047805 | 226.969482 |
| EB25M | OU error | 858.16834 | 241.41319 |
| EB25M | OUf anisotropic error | 1366.2005 | 1109.95766 |
| EB25M | OUf error | 1408.74182 | 1115.40222 |
| EB26F | OUF error | 0 | 207.271199 |
| EB26F | OUF anisotropic error | 0.48200139 | 200.550782 |
| EB26F | OUf error | 191.711969 | 376.518371 |
| EB26F | OU error | 239.258816 | 158.635248 |
| EB27M | OUF anisotropic error | 0 | 400.581726 |
| EB27M | OUF error | 84.6686144 | 401.281251 |
| EB27M | OUf anisotropic error | 493.455498 | 875.429644 |
| EB27M | OUf error | 582.282805 | 984.432797 |
| EB27M | OU anisotropic error | 990.599155 | 247.49531 |
| EB27M | OU error | 1106.25482 | 241.730424 |
| EB28F | OUF anisotropic error | 0 | 287.924319 |
| EB28F | OUF error | 86.9345128 | 1.1358E-05 |
| EB28F | OU anisotropic error | 665.148144 | 716.236868 |
| EB28F | OU error | 819.764554 | 779.416939 |
| EB28F | OUf anisotropic error | 1389.13653 | 926.276951 |
| EB28F | OUf error | 1486.69392 | 931.810869 |
| EB29F | OUF anisotropic error | 0 | 148.452415 |
| EB29F | OUF error | 142.035027 | 148.303769 |
| EB29F | OUf anisotropic error | 278.084348 | 310.854537 |
| EB29F | OU anisotropic error | 307.343881 | 108.743277 |
| EB29F | OUf error | 420.435395 | 311.568507 |
| EB29F | OU error | 518.517677 | 104.062599 |
| EB30F | OUF anisotropic error | 0 | 302.589688 |
| EB30F | OUF error | 60.942169 | 294.384729 |
| EB30F | OU anisotropic error | 594.264174 | 217.913267 |
| EB30F | OU error | 672.050383 | 219.338015 |
| EB30F | OUf anisotropic error | 1084.93352 | 903.732063 |
| EB30F | OUf error | 1204.02238 | 908.645875 |
| EB31M | OUF anisotropic error | 0 | 2.74836654 |
| EB31M | OUF error | 5.1326478 | 2.87176609 |
| EB31M | OU anisotropic error | 79.5363692 | 2.77146106 |
| EB31M | OU error | 93.9170286 | 1.84306698 |
| EB31M | OUf error | 153.631251 | 37.757576 |
| EB31M | OUf anisotropic error | 156.170278 | 36.7073003 |
| EB32F | OUF anisotropic error | 0 | 7.54764095 |
| EB32F | OU anisotropic error | 157.115552 | 5.5992515 |
| EB32F | OU error | 1108.24424 | 6.97035207 |
| EB32F | OUf anisotropic error | 1201.64709 | 313.756726 |
| EB32F | OUf error | 2022.0033 | 152.977395 |
| EB32F | OUF error | 2232.59859 | 8.89525926 |
| EB34M | OUF error | 0 | 22.8967834 |
| EB34M | OUF anisotropic error | 3.86652618 | 21.3646858 |
| EB34M | OU error | 48.3743651 | 27.0118472 |
| EB34M | OUf error | 49.7916436 | 55.3639671 |
| EB35M | OUF anisotropic error | 0 | 35.2017299 |
| EB35M | OU anisotropic error | 116.165962 | 29.2128295 |
| EB35M | OU error | 198.359313 | 31.6198532 |
| EB35M | OUF error | 693.731182 | 3.50803836 |
| EB35M | OUf anisotropic error | 1329.75599 | 331.774599 |
| EB35M | OUf error | 1358.04304 | 361.239247 |
| EB36M | OUF anisotropic error | 0 | 352.537224 |
| EB36M | OU anisotropic error | 113.277148 | 289.93194 |
| EB36M | OUF error | 156.10967 | 322.050251 |
| EB36M | OU error | 256.019904 | 351.291252 |
| EB36M | OUf anisotropic error | 2055.31608 | 1118.49415 |
| EB36M | OUf error | 2271.48375 | 1053.2145 |
| EB37M | OUF anisotropic error | 0 | 552.485952 |
| EB37M | OUF error | 200.610135 | 286.697126 |
| EB37M | OU anisotropic error | 801.37631 | 994.568687 |
| EB37M | OU error | 1076.95508 | 1081.42864 |
| EB37M | OUf anisotropic error | 3714.84389 | 2043.24529 |
| EB37M | OUf error | 3998.00596 | 2063.39698 |
| EB39M | OUF anisotropic error | 0 | 9.06890091 |
| EB39M | OUF error | 45.4741459 | 10.0307193 |
| EB39M | OUf anisotropic error | 269.925725 | 76.1196192 |
| EB39M | OUf error | 425.208417 | 215.5285 |
| EB39M | OU anisotropic error | 1119.03723 | 4.89604128 |
| EB39M | OU error | 1245.59081 | 5.75434212 |
| EB40F | OUF anisotropic error | 0 | 173.659595 |
| EB40F | OU anisotropic error | 225.908965 | 135.01342 |
| EB40F | OUF error | 397.310011 | 161.175279 |
| EB40F | OU error | 710.725847 | 125.137974 |
| EB40F | OUf anisotropic error | 1550.26086 | 820.89163 |
| EB40F | OUf error | 3594.48986 | 2935.08364 |
| EB41F | OUF error | 0 | 39.4687329 |
| EB41F | OUF anisotropic error | 4.02986919 | 107.563716 |
| EB41F | OU error | 118.793489 | 35.1895749 |
| EB41F | OUf error | 1501.12709 | 372.834049 |
| EB63F | OUF anisotropic error | 0 | 616.322607 |
| EB63F | OUF error | 80.868347 | 678.425469 |
| EB63F | OU anisotropic error | 1062.43257 | 483.240297 |
| EB63F | OU error | 1133.83384 | 454.116961 |
| EB63F | OUf anisotropic error | 1321.39677 | 2530.0068 |
| EB63F | OUf error | 5756.45625 | 1225.01232 |
| EB64F | OUF anisotropic error | 0 | 382.709086 |
| EB64F | OUF error | 56.9802525 | 375.833644 |
| EB64F | OUf anisotropic error | 1206.03916 | 984.775291 |
| EB64F | OU anisotropic error | 1277.85123 | 246.431733 |
| EB64F | OU error | 1382.46663 | 236.035829 |
| EB64F | OUf error | 1895.11223 | 2418.95299 |
| EB65M | OUF anisotropic error | 0 | 8.46650946 |
| EB65M | OU anisotropic error | 9.13256449 | 9.04007155 |
| EB65M | OUF error | 19.9863072 | 5.34707064 |
| EB65M | OU error | 24.6370376 | 11.1695592 |
| EB65M | OUf anisotropic error | 80.3807444 | 32.6040647 |
| EB65M | OUf error | 85.8576155 | 31.329462 |
| EB66M | OUF anisotropic error | 0 | 16.2562032 |
| EB66M | OUF error | 115.610286 | 13.5046838 |
| EB66M | OU anisotropic error | 371.191368 | 16.7235405 |
| EB66M | OU error | 449.355064 | 16.8337683 |
| EB66M | OUf anisotropic error | 2608.84314 | 381.507131 |
| EB66M | OUf error | 2675.66419 | 396.572253 |
| EB67M | OUF anisotropic error | 0 | 2.77168244 |
| EB67M | OUF error | 56.6914629 | 18.6781581 |
| EB67M | OU anisotropic error | 986.409163 | 4600.64238 |
| EB67M | OU error | 1000.40546 | 60.6394007 |
| EB67M | OUf anisotropic error | 2590.06237 | 334.69282 |
| EB67M | OUf error | 2609.05868 | 367.208175 |
| EB68M | OUF anisotropic error | 0 | 481.512598 |
| EB68M | OUf error | 216.917572 | 453.934687 |
| EB68M | OU anisotropic error | 649.681569 | 339.865666 |
| EB68M | OUF error | 934.766275 | 304.180661 |
| EB68M | OU error | 1656.03617 | 42.9269694 |
| EB68M | OUf anisotropic error | 1911.12038 | 1545.89923 |
| EB69F | OUF anisotropic error | 0 | 462.631148 |
| EB69F | OUF error | 7.75561121 | 468.917111 |
| EB69F | OU anisotropic error | 463.193064 | 345.127525 |
| EB69F | OU error | 471.156776 | 348.369383 |
| EB69F | OUf anisotropic error | 579.70091 | 952.98196 |
| EB69F | OUf error | 582.111315 | 965.775699 |
| EB70M | OUF anisotropic error | 0 | 45.0384476 |
| EB70M | OU anisotropic error | 63.5560807 | 47.924683 |
| EB70M | OUF error | 95.285125 | 55.4673483 |
| EB70M | OU error | 166.96348 | 43.8325734 |
| EB70M | OUf anisotropic error | 212.897741 | 117.864412 |
| EB70M | OUf error | 259.491142 | 125.166652 |
| EB71M | OU error | 0 | 1.54696189 |
| EB71M | OUF error | 155.019858 | 1.1362E-15 |
| EB71M | OUf error | 1821.02655 | 302.873436 |
| EB71M | OUF anisotropic error | 1936.89825 | 380.593001 |
| EB71M | IID error | 58074.4216 | 5760.99998 |
| EB72M | OUF anisotropic error | 0 | 37.767875 |
| EB72M | OUF error | 549.097573 | 46.2710121 |
| EB72M | OU anisotropic error | 599.569676 | 24.7965438 |
| EB72M | OU error | 1185.8909 | 34.7325094 |
| EB72M | OUf anisotropic error | 1383.1282 | 365.466581 |
| EB72M | OUf error | 1772.2315 | 395.114734 |
| EB73F | OUF anisotropic error | 0 | 34.1001735 |
| EB73F | OUF error | 3.16243549 | 34.7163963 |
| EB73F | OU error | 897.463817 | 27.7981975 |
| EB73F | OU anisotropic error | 900.292113 | 20.959486 |
| EB73F | OUf anisotropic error | 2055.59863 | 372.688812 |
| EB73F | OUf error | 2081.60738 | 408.124751 |
| EB76M | OUF anisotropic error | 0 | 22.2172261 |
| EB76M | OUF error | 188.755419 | 19.8701871 |
| EB76M | OU anisotropic error | 918.188065 | 10.1121861 |
| EB76M | OUf anisotropic error | 1064.3307 | 258.707995 |
| EB76M | OUf error | 1236.96193 | 259.597574 |
| EB76M | OU error | 1293.33787 | 8.61043816 |
| EB77M | OUF anisotropic error | 0 | 198.463978 |
| EB77M | OUF error | 7.53608087 | 200.210389 |
| EB77M | OU anisotropic error | 122.460237 | 164.192511 |
| EB77M | OU error | 126.564624 | 219.950193 |
| EB77M | OUf anisotropic error | 1403.97879 | 1815.96334 |
| EB77M | OUf error | 1658.74636 | 2026.42104 |
| EB78M | OUF error | 0 | 73.7433182 |
| EB78M | OUF anisotropic error | 3.71375247 | 72.8717464 |
| EB78M | OU error | 451.193568 | 53.8609903 |
| EB78M | OUf error | 1229.94001 | 463.829157 |
| EB79M | OUF anisotropic error | 0 | 190.110656 |
| EB79M | OUF error | 202.922696 | 194.313483 |
| EB79M | OU anisotropic error | 417.012121 | 138.606065 |
| EB79M | OU error | 630.544852 | 135.847431 |
| EB79M | OUf anisotropic error | 1390.11795 | 1384.29086 |
| EB79M | OUf error | 3482.57409 | 3164.20019 |
| EB80M | OUF anisotropic error | 0 | 478.303896 |
| EB80M | OUF error | 102.659876 | 478.238778 |
| EB80M | OUf anisotropic error | 425.185181 | 805.586558 |
| EB80M | OU anisotropic error | 480.757579 | 332.24715 |
| EB80M | OUf error | 509.704072 | 795.513142 |
| EB80M | OU error | 584.79483 | 345.568705 |
| EB81F | OUF anisotropic error | 0 | 41.8252774 |
| EB81F | OUF error | 50.8883494 | 50.4192232 |
| EB81F | OU anisotropic error | 465.077305 | 102.212745 |
| EB81F | OU error | 488.268555 | 97.7408948 |
| EB81F | OUf error | 1387.97545 | 861.61539 |
| EB81F | OUf anisotropic error | 3065.19764 | 999.41119 |
| EB82F | OUF anisotropic error | 0 | 21.0493208 |
| EB82F | OUF error | 48.3125116 | 22.6810459 |
| EB82F | OU anisotropic error | 665.112129 | 10.9762446 |
| EB82F | OU error | 738.571914 | 11.7449171 |
| EB82F | OUf anisotropic error | 876.176231 | 227.02234 |
| EB82F | OUf error | 914.498369 | 241.326696 |
| EB83F | OUF anisotropic error | 0 | 16.3398357 |
| EB83F | OUF error | 20.2530244 | 16.5519246 |
| EB83F | OU anisotropic error | 293.837258 | 11.9562339 |
| EB83F | OU error | 352.012902 | 11.7752351 |
| EB83F | OUf anisotropic error | 1475.01966 | 253.981243 |
| EB83F | OUf error | 1477.8164 | 256.872248 |
| HB727054F | OUF anisotropic error | 0 | 317.928445 |
| HB727054F | OUF error | 21.7028247 | 340.506132 |
| HB727054F | OUf anisotropic error | 528.130949 | 756.896618 |
| HB727054F | OUf error | 568.862862 | 766.005486 |
| HB727054F | OU anisotropic error | 917.374436 | 943.859124 |
| HB727054F | OU error | 977.267385 | 990.659596 |
| HB729230M | OUF anisotropic error | 0 | 30.5148342 |
| HB729230M | OUF error | 3.58275537 | 53.3478392 |
| HB729230M | OU anisotropic error | 183.079433 | 21.1821444 |
| HB729230M | OU error | 186.959638 | 24.0544365 |
| HB729230M | OUf anisotropic error | 574.246066 | 184.600912 |
| HB729230M | OUf error | 589.067979 | 195.396943 |
| HB729232F | OU anisotropic error | 0 | 2.11120285 |
| HB729232F | OU error | 10.8774344 | 4.58287243 |
| HB729232F | OUF anisotropic error | 56.8220944 | 2789.46537 |
| HB729232F | OUF error | 1648.84936 | 4.00462123 |
| HB729232F | OUf anisotropic error | 1793.81262 | 172.844645 |
| HB729232F | OUf error | 1825.62997 | 179.451388 |
| HB729232F | IID anisotropic error | 44331.8292 | 3581.99999 |
| HB729233M | OUF anisotropic error | 0 | 92.7950108 |
| HB729233M | OUF error | 92.4903341 | 448.800513 |
| HB729233M | OU anisotropic error | 859.820224 | 60.4274869 |
| HB729233M | OU error | 906.798545 | 64.5659063 |
| HB729233M | OUf anisotropic error | 1103.84663 | 472.413004 |
| HB729233M | OUf error | 1143.24779 | 467.978118 |
| HB729234F | OUF anisotropic error | 0 | 294.523326 |
| HB729234F | OUF error | 14.4018256 | 294.999538 |
| HB729234F | OU anisotropic error | 578.795895 | 198.97569 |
| HB729234F | OU error | 603.90199 | 198.250147 |
| HB729234F | OUf anisotropic error | 765.084993 | 818.635241 |
| HB729234F | OUf error | 781.203288 | 820.092597 |
| KRB1M | OU anisotropic | 0 | 94.1112793 |
| KRB1M | OUF anisotropic | 2.09389221 | 98.6727748 |
| KRB1M | OUf anisotropic | 12.8578022 | 116.112509 |
| KRB1M | OUF | 55.935584 | 87.4294646 |
| KRB1M | OU | 57.2524725 | 82.2560204 |
| KRB1M | IID anisotropic | 116.981583 | 184 |
| KRB2M | OUf anisotropic | 0 | 175.555625 |
| KRB2M | OUF anisotropic | 2.06669913 | 175.555637 |
| KRB2M | OUf | 4.78138331 | 176.611548 |
| KRB2M | OUF | 6.82344247 | 176.611548 |
| KRB2M | OU anisotropic | 47.6812081 | 152.292564 |
| KRB2M | IID anisotropic | 395.191874 | 337 |
| KRB2M | OUΩ anisotropic | 398.537275 | 0.00046038 |
| KRB3F | OU anisotropic | 0 | 212.428378 |
| KRB3F | OUF anisotropic | 2.04190456 | 216.855515 |
| KRB3F | OU | 34.9333651 | 278.664852 |
| KRB3F | OUF | 36.9605077 | 281.746067 |
| KRB3F | IID anisotropic | 201.992963 | 404 |
| KRB3F | OUf anisotropic | 213.12279 | 390.93852 |
| KRB4F | OU anisotropic | 0 | 766.165427 |
| KRB4F | OUF anisotropic | 2.01322781 | 770.826254 |
| KRB4F | OU | 249.979825 | 856.209561 |
| KRB4F | OUF | 251.987332 | 860.268027 |
| KRB4F | IID anisotropic | 573.709385 | 1259 |
| KRB4F | OUf anisotropic | 575.719656 | 1258.9989 |
| KRB5F | OUf anisotropic | 0 | 343.780519 |
| KRB5F | OUF anisotropic | 2.0398673 | 343.78052 |
| KRB5F | OUf | 8.94396539 | 343.495698 |
| KRB5F | OUF | 10.9691969 | 343.4957 |
| KRB5F | OU anisotropic | 48.6206664 | 314.782612 |
| KRB5F | IID anisotropic | 468.630823 | 559 |
| KRB5F | OUΩ anisotropic | 478.366757 | 0.00295173 |
| KRB6M | OU anisotropic | 0 | 170.8472 |
| KRB6M | OUF anisotropic | 1.33766931 | 175.259623 |
| KRB6M | OU | 122.558599 | 150.004466 |
| KRB6M | OUF | 122.798368 | 157.513389 |
| KRB6M | OUf anisotropic | 123.177607 | 326.131183 |
| KRB6M | IID anisotropic | 1001.39441 | 631 |
| KRB8M | OUF anisotropic | 0 | 28.2676771 |
| KRB8M | OUF | 17.5065968 | 27.1690288 |
| KRB8M | OU anisotropic | 163.495497 | 15.8743339 |
| KRB8M | OUf anisotropic | 234.182663 | 143.917974 |
| KRB9M | OUF anisotropic | 0 | 311.689537 |
| KRB9M | OUF | 17.915897 | 315.476997 |
| KRB9M | OU anisotropic | 18.506574 | 309.105206 |
| KRB9M | OUf anisotropic | 43.5776693 | 349.555595 |
| LB726981M | OUF anisotropic error | 0 | 59.747057 |
| LB726981M | OUF error | 222.655239 | 55.0026168 |
| LB726981M | OU anisotropic error | 470.090704 | 50.9481921 |
| LB726981M | OU error | 769.342909 | 41.033894 |
| LB726981M | OUf anisotropic error | 1675.21485 | 471.777818 |
| LB726981M | OUf error | 1975.97106 | 613.100514 |
| LB727047M | OUF anisotropic error | 0 | 394.777679 |
| LB727047M | OUF error | 377.821145 | 381.607569 |
| LB727047M | OU anisotropic error | 461.297247 | 289.652312 |
| LB727047M | OUf anisotropic error | 674.287082 | 945.981817 |
| LB727047M | OU error | 1006.63037 | 585.926906 |
| LB727047M | OUf error | 1074.50429 | 2292.53496 |
| LB727051F | OUF anisotropic error | 0 | 3.45659056 |
| LB727051F | OUF error | 0.26799322 | 2.83390146 |
| LB727051F | OU anisotropic error | 1.23385999 | 3.01716657 |
| LB727051F | OU error | 1.9849798 | 3.21240544 |
| LB727051F | OUf anisotropic error | 32.0482076 | 9.30568552 |
| LB727051F | OUf error | 34.0144554 | 15.3844953 |
| LB727052M | OUF anisotropic error | 0 | 363.479229 |
| LB727052M | OU anisotropic error | 567.22525 | 483.866792 |
| LB727052M | OUf anisotropic error | 920.654246 | 1502.3812 |
| LB727052M | OUF error | 978.663417 | 651.988184 |
| LB727052M | OU error | 1631.82112 | 463.591377 |
| LB727052M | OUf error | 2028.32372 | 1532.93705 |
| YB7M | OUF anisotropic error | 0 | 79.9946125 |
| YB7M | OUF error | 71.4753036 | 72.8545097 |
| YB7M | OU anisotropic error | 141.074254 | 47.9700158 |
| YB7M | OU error | 216.3899 | 49.1059389 |
| YB7M | OUf anisotropic error | 226.363333 | 202.61241 |
| YB7M | OUf error | 273.063637 | 201.176656 |
| EB01F | IID | 0 | 40 |
| EB01F | IID anisotropic | 4.12148356 | 40 |
| EB02M | IID | 0 | 27 |
| EB02M | IID anisotropic | 1.16325063 | 27 |
| EB03F | IID | 0 | 103 |
| EB03F | IID anisotropic | 0.30158643 | 103 |
| EB04F | IID anisotropic | 0 | 99 |
| EB04F | IID | 3.53670692 | 99 |
| EB07M | IID | 0 | 108 |
| EB07M | IID anisotropic | 2.64455146 | 108 |
| EB09F | IID | 0 | 104 |
| EB09F | IID anisotropic | 2.91749815 | 104 |
| EB10M | IID anisotropic | 0 | 102 |
| EB10M | IID | 3.74776269 | 102 |
| EB11M | IID anisotropic | 0 | 94 |
| EB11M | IID | 6.40544905 | 94 |
| EB12F | IID anisotropic | 0 | 57 |
| EB12F | IID | 16.5668252 | 57 |
| EB13M | IID | 0 | 58 |
| EB13M | IID anisotropic | 1.6570041 | 58 |
| EB14M | IID anisotropic | 0 | 89 |
| EB14M | IID | 7.40288516 | 89 |
| LB028M | IID anisotropic | 0 | 56 |
| LB028M | IID | 23.979372 | 56 |
| LB040U | IID anisotropic | 0 | 97 |
| LB040U | IID | 127.15011 | 97 |
| LB058U | IID anisotropic | 0 | 59 |
| LB058U | IID | 14.3111519 | 59 |
| LB064M | IID anisotropic | 0 | 62 |
| LB064M | IID | 31.0662079 | 62 |
| LB070M | IID anisotropic | 0 | 65 |
| LB070M | IID | 17.2121191 | 65 |
| LB071M | IID anisotropic | 0 | 84 |
| LB071M | IID | 100.066971 | 84 |
| LB076U | IID anisotropic | 0 | 134 |
| LB076U | IID | 88.2298679 | 134 |
| LB078U | IID anisotropic | 0 | 176 |
| LB078U | IID | 13.7490413 | 176 |
| LB106M | IID anisotropic | 0 | 91 |
| LB106M | IID | 19.1512802 | 91 |
| LB119F | IID anisotropic | 0 | 34 |
| LB119F | IID | 22.8994993 | 34 |
| LB122M | IID anisotropic | 0 | 36 |
| LB122M | IID | 13.4360277 | 36 |
| LB124M | IID anisotropic | 0 | 31 |
| LB124M | IID | 2.49188843 | 31 |
| LB129M | IID anisotropic | 0 | 83 |
| LB129M | IID | 14.8518825 | 83 |
| LB130F | IID anisotropic | 0 | 101 |
| LB130F | IID | 1.37190544 | 101 |
| LB134F | IID anisotropic | 0 | 27 |
| LB134F | IID | 5.48586913 | 27 |
| LB140F | IID anisotropic | 0 | 32 |
| LB140F | IID | 21.0716391 | 32 |
| LB144F | IID anisotropic | 0 | 32 |
| LB144F | IID | 1.90585073 | 32 |
| LB146M | IID anisotropic | 0 | 41 |
| LB146M | IID | 78.6825906 | 41 |
| LB148M | IID anisotropic | 0 | 51 |
| LB148M | IID | 7.47435602 | 51 |
| LB152M | IID anisotropic | 0 | 33 |
| LB152M | IID | 17.9720455 | 33 |
| LB154M | IID anisotropic | 0 | 26 |
| LB154M | IID | 2.70521162 | 26 |
| LB157F | IID anisotropic | 0 | 47 |
| LB157F | IID | 13.6611338 | 47 |
| LB163M | IID anisotropic | 0 | 25 |
| LB163M | IID | 22.1412055 | 25 |
| SAB04F | IID anisotropic | 0 | 96 |
| SAB04F | IID | 6.46690764 | 96 |
| SAB05F | IID anisotropic | 0 | 95 |
| SAB05F | IID | 7.67682593 | 95 |
| SAB06M | IID anisotropic | 0 | 76 |
| SAB06M | IID | 4.23183663 | 76 |
| SAB07M | IID | 0 | 43 |
| SAB07M | IID anisotropic | 4.14519814 | 43 |
| SAB08M | IID anisotropic | 0 | 102 |
| SAB08M | IID | 6.7603 | 102 |
| SAB10F | IID anisotropic | 0 | 92 |
| SAB10F | IID | 22.1650468 | 92 |
| SAB13M | IID anisotropic | 0 | 76 |
| SAB13M | IID | 30.4031208 | 76 |
| SAB15M | IID | 0 | 62 |
| SAB15M | IID anisotropic | 4.19499644 | 62 |
| SAB19F | IID | 0 | 66 |
| SAB19F | IID anisotropic | 1.19323443 | 66 |
| WB07F | IID anisotropic | 0 | 350 |
| WB07F | IID | 301.802163 | 350 |
| WB12M | IID anisotropic | 0 | 459 |
| WB12M | IID | 823.319927 | 459 |
| WB32F | IID anisotropic | 0 | 444 |
| WB32F | IID | 11.4411572 | 444 |
| WB39F | IID anisotropic | 0 | 325 |
| WB39F | IID | 209.573861 | 325 |
| WB40F | IID anisotropic | 0 | 73 |
| WB40F | IID | 43.497218 | 73 |
| WB42M | IID anisotropic | 0 | 201 |
| WB42M | IID | 6.40816685 | 201 |
| WB44M | IID | 0 | 28 |
| WB44M | IID anisotropic | 4.57958504 | 28 |
| WB47M | IID anisotropic | 0 | 224 |
| WB47M | IID | 23.0280228 | 224 |
| WB48F | IID anisotropic | 0 | 35 |
| WB48F | IID | 15.2205266 | 35 |
| WB53M | IID | 0 | 46 |
| WB53M | IID anisotropic | 2.05236815 | 46 |
| WB55M | IID anisotropic | 0 | 52 |
| WB55M | IID | 7.20424519 | 52 |
| YB1F | IID anisotropic | 0 | 26 |
| YB1F | IID | 5.42498385 | 26 |
| EB08M | OUF anisotropic | 0 | 170.43546 |
| EB08M | OUf anisotropic | 59.9609441 | 196.093425 |
| EB08M | OUF | 88.0178181 | 178.441841 |
| EB08M | OU anisotropic | 181.370387 | 148.765464 |
| EB15F | OUF | 0 | 275.841962 |
| EB15F | OUF anisotropic | 1.64764296 | 273.054481 |
| EB15F | OUf | 492.189525 | 517.419599 |
| EB15F | OUf anisotropic | 495.680258 | 510.231764 |
| EB15F | OU anisotropic | 826.277648 | 176.15061 |
| EB15F | OU | 829.967855 | 176.161404 |
| EB16M | OUF anisotropic | 0 | 11.6628713 |
| EB16M | OUF | 232.314703 | 9.46656785 |
| EB16M | OU anisotropic | 667.202483 | 6.4123415 |
| EB16M | OUf anisotropic | 2498.16135 | 363.955326 |
| EB18M | OUF anisotropic | 0 | 173.099952 |
| EB18M | OUF | 35.7443196 | 173.075712 |
| EB18M | OU anisotropic | 95.2962499 | 160.286491 |
| EB18M | OUf anisotropic | 751.122624 | 312.870361 |

Supplemental Data SD3. Monitoring durations and number of individuals by sex, collar type, and decade. Rows with empty cells are averaged across levels of the missing factor.

| Sex | Collar Type | Decade | n | Mean ± SD | Range |
| --- | --- | --- | --- | --- | --- |
| F | GPS | 2010s | 5 | 117.53 ± 45.2 | 68 - 177 |
| F | GPS | 2020s | 24 | 206.52 ± 99.8 | 54 - 379 |
| F | VHF | 1980s | 2 | 333.18 ± 189.6 | 199 - 467 |
| F | VHF | 1990s | 12 | 586.98 ± 454.6 | 132 - 1513 |
| F | VHF | 2000s | 2 | 672.14 ± 742.2 | 147 - 1197 |
| F | VHF | 2010s | 5 | 409.05 ± 147.6 | 158 - 522 |
| M | GPS | 2010s | 8 | 101.5 ± 42.3 | 44 - 187 |
| M | GPS | 2020s | 28 | 195.77 ± 98 | 30 - 375 |
| M | VHF | 1980s | 9 | 497.51 ± 384.6 | 213 - 1410 |
| M | VHF | 1990s | 11 | 418.37 ± 445.1 | 155 - 1702 |
| M | VHF | 2000s | 4 | 498.94 ± 289.1 | 128 - 783 |
| M | VHF | 2010s | 6 | 297.24 ± 119.8 | 136 - 460 |
| U | VHF | 1980s | 4 | 445.08 ± 261.1 | 182 - 743 |
| F | GPS |  | 29 | 191.18 ± 98.2 | 54 - 379 |
| F | VHF |  | 21 | 528.55 ± 398.6 | 132 - 1513 |
| M | GPS |  | 36 | 174.82 ± 96.7 | 30 - 375 |
| M | VHF |  | 30 | 428.63 ± 355 | 128 - 1702 |
| U | VHF |  | 4 | 445.08 ± 261.1 | 182 - 743 |
| F |  | 1980s | 2 | 333.18 ± 189.6 | 199 - 467 |
| F |  | 1990s | 12 | 586.98 ± 454.6 | 132 - 1513 |
| F |  | 2000s | 2 | 672.14 ± 742.2 | 147 - 1197 |
| F |  | 2010s | 10 | 263.29 ± 184.9 | 68 - 522 |
| F |  | 2020s | 24 | 206.52 ± 99.8 | 54 - 379 |
| M |  | 1980s | 9 | 497.51 ± 384.6 | 213 - 1410 |
| M |  | 1990s | 11 | 418.37 ± 445.1 | 155 - 1702 |
| M |  | 2000s | 4 | 498.94 ± 289.1 | 128 - 783 |
| M |  | 2010s | 14 | 185.39 ± 128.8 | 44 - 460 |
| M |  | 2020s | 28 | 195.77 ± 98 | 30 - 375 |
| U |  | 1980s | 4 | 445.08 ± 261.1 | 182 - 743 |
|  | GPS | 2010s | 13 | 107.67 ± 42.3 | 44 - 187 |
|  | GPS | 2020s | 52 | 200.73 ± 98 | 30 - 379 |
|  | VHF | 1980s | 15 | 461.62 ± 324 | 182 - 1410 |
|  | VHF | 1990s | 23 | 506.34 ± 448.1 | 132 - 1702 |
|  | VHF | 2000s | 6 | 556.67 ± 410.3 | 128 - 1197 |
|  | VHF | 2010s | 11 | 348.06 ± 138.9 | 136 - 522 |
| F |  |  | 50 | 332.88 ± 314.1 | 54 - 1513 |
| M |  |  | 66 | 290.19 ± 278.3 | 30 - 1702 |
| U |  |  | 4 | 445.08 ± 261.1 | 182 - 743 |
|  | GPS |  | 65 | 182.12 ± 96.9 | 30 - 379 |
|  | VHF |  | 55 | 467.98 ± 364.2 | 128 - 1702 |
|  |  | 1980s | 15 | 461.62 ± 324 | 182 - 1410 |
|  |  | 1990s | 23 | 506.34 ± 448.1 | 132 - 1702 |
|  |  | 2000s | 6 | 556.67 ± 410.3 | 128 - 1197 |
|  |  | 2010s | 24 | 217.85 ± 155.9 | 44 - 522 |
|  |  | 2020s | 52 | 200.73 ± 98 | 30 - 379 |

Supplemental Data SD4: Estimate of population-level home range size (in km^2^) for bobcats collared from 1981 to 2024 in South Texas. Lower and upper limits are lower and upper 95% confidence limits.

| Group | Sample Size | Lower Limit | Estimate | Upper Limit |
| --- | --- | --- | --- | --- |
| Residents | 103 | 6.50 | 8.04 | 9.82 |
| All females | 42 | 3.37 | 4.20 | 5.16 |
| All males | 57 | 8.28 | 11.00 | 14.32 |
| 1980s (all) | 14 | 5.61 | 9.37 | 14.73 |
| 1980s (F) | 2 | 5.19 | 6.71 | 8.42 |
| 1980s (M) | 8 | 7.39 | 11.39 | 16.78 |
| 1990s (all) | 22 | 4.83 | 7.78 | 11.89 |
| 1990s (F) | 11 | 3.15 | 5.33 | 8.48 |
| 1990s (M) | 11 | 4.71 | 10.26 | 19.65 |
| 2000s (all) | 6 | 3.80 | 7.36 | 12.91 |
| 2000s (F) | 2 | 2.24 | 2.93 | 3.70 |
| 2000s (M) | 4 | 5.04 | 9.53 | 16.36 |
| 2010s (all) | 23 | 5.56 | 9.61 | 15.50 |
| 2010s (F) | 10 | 2.63 | 4.03 | 5.91 |
| 2010s (M) | 13 | 6.28 | 14.00 | 27.23 |
| 2020s (all) | 38 | 4.90 | 6.87 | 9.37 |
| 2020s (F) | 17 | 2.48 | 3.39 | 4.53 |
| 2020s (M) | 21 | 6.20 | 9.70 | 14.47 |
| VHF (all) | 53 | 6.91 | 9.21 | 12.02 |
| VHF (F) | 20 | 3.77 | 5.28 | 7.20 |
| VHF (M) | 29 | 8.51 | 12.29 | 17.18 |
| GPS (all) | 50 | 4.99 | 6.78 | 8.99 |
| GPS (F) | 22 | 2.50 | 3.20 | 4.04 |
| GPS (M) | 28 | 6.27 | 9.66 | 14.23 |

Supplemental Data SD5: Estimates of percent home range overlap between conspecific bobcats. Only individuals that were monitored at the same time in the same area were compared.

| Comparison | Collar Type | Lower limit | Estimate | Upper limit | High* |
| --- | --- | --- | --- | --- | --- |
| EB01F-EB02M | VHF | 36.57% | 48.03% | 60.45% | Yes |
| EB03F-EB04F | VHF | 0.00% | 0.00% | 0.02% | No |
| EB07M-EB09F | VHF | 1.34% | 2.40% | 4.11% | No |
| EB07M-EB10M | VHF | 9.15% | 13.57% | 19.44% | No |
| EB07M-EB11M | VHF | 68.52% | 76.84% | 84.42% | Yes |
| EB07M-EB12F | VHF | 25.98% | 35.30% | 46.12% | Yes |
| EB07M-EB13M | VHF | 0.04% | 0.14% | 0.47% | No |
| EB07M-EB14M | VHF | 0.00% | 0.00% | 0.00% | No |
| EB07M-EB15F | VHF | 0.00% | 0.00% | 0.00% | No |
| EB09F-EB10M | VHF | 1.76% | 3.01% | 4.94% | No |
| EB09F-EB11M | VHF | 5.32% | 8.36% | 12.65% | No |
| EB09F-EB12F | VHF | 48.59% | 57.77% | 67.10% | Yes |
| EB09F-EB13M | VHF | 0.00% | 0.00% | 0.00% | No |
| EB09F-EB14M | VHF | 0.00% | 0.00% | 0.00% | No |
| EB09F-EB15F | VHF | 0.00% | 0.00% | 0.00% | No |
| EB10M-EB11M | VHF | 55.82% | 64.62% | 73.26% | Yes |
| EB10M-EB12F | VHF | 7.35% | 12.59% | 20.30% | No |
| EB10M-EB13M | VHF | 0.00% | 0.01% | 0.06% | No |
| EB10M-EB14M | VHF | 0.00% | 0.00% | 0.00% | No |
| EB10M-EB15F | VHF | 0.00% | 0.00% | 0.00% | No |
| EB11M-EB12F | VHF | 34.06% | 43.71% | 54.31% | Yes |
| EB11M-EB13M | VHF | 0.00% | 0.00% | 0.02% | No |
| EB11M-EB14M | VHF | 0.00% | 0.00% | 0.00% | No |
| EB11M-EB15F | VHF | 0.00% | 0.00% | 0.00% | No |
| EB12F-EB13M | VHF | 0.00% | 0.00% | 0.00% | No |
| EB12F-EB14M | VHF | 0.00% | 0.00% | 0.00% | No |
| EB12F-EB15F | VHF | 0.00% | 0.00% | 0.00% | No |
| EB13M-EB14M | VHF | 50.69% | 59.41% | 68.19% | Yes |
| EB13M-EB15F | VHF | 33.07% | 39.14% | 45.69% | Yes |
| EB14M-EB15F | VHF | 84.50% | 89.99% | 94.44% | Yes |
| LB028M-LB040U | VHF | 6.81% | 10.70% | 16.12% | No |
| LB028M-LB058U | VHF | 34.68% | 44.84% | 55.95% | Yes |
| LB028M-LB064M | VHF | 85.50% | 90.71% | 94.92% | Yes |
| LB028M-LB070M | VHF | 27.02% | 36.48% | 47.39% | Yes |
| LB040U-LB058U | VHF | 47.12% | 54.15% | 61.36% | Yes |
| LB040U-LB064M | VHF | 1.19% | 2.66% | 5.49% | No |
| LB040U-LB070M | VHF | 38.95% | 46.13% | 53.75% | Yes |
| LB058U-LB064M | VHF | 22.44% | 31.89% | 43.26% | Yes |
| LB058U-LB070M | VHF | 85.95% | 93.49% | 98.31% | Yes |
| LB064M-LB070M | VHF | 15.34% | 22.32% | 31.17% | Yes |
| LB071M-LB076U | VHF | 25.55% | 29.16% | 33.05% | Yes |
| LB071M-LB078U | VHF | 0.00% | 0.00% | 0.00% | No |
| LB071M-LB146M | VHF | 0.00% | 0.00% | 0.00% | No |
| LB071M-LB148M | VHF | 0.00% | 0.00% | 0.00% | No |
| LB071M-LB152M | VHF | 36.66% | 44.73% | 53.40% | Yes |
| LB071M-LB154M | VHF | 0.00% | 0.02% | 0.07% | No |
| LB071M-LB106M | VHF | 51.68% | 56.94% | 62.26% | Yes |
| LB071M-LB119F | VHF | 19.08% | 25.53% | 33.23% | Yes |
| LB071M-LB122M | VHF | 35.12% | 42.83% | 51.15% | Yes |
| LB071M-LB124M | VHF | 0.00% | 0.01% | 0.13% | No |
| LB071M-LB129M | VHF | 32.89% | 38.44% | 44.41% | Yes |
| LB071M-LB130F | VHF | 24.71% | 29.37% | 34.52% | Yes |
| LB071M-LB134F | VHF | 0.03% | 0.08% | 0.20% | No |
| LB071M-LB140F | VHF | 0.02% | 0.11% | 0.56% | No |
| LB071M-LB144F | VHF | 0.01% | 0.06% | 0.21% | No |
| LB071M-LB157F | VHF | 13.59% | 17.53% | 22.23% | Yes |
| LB071M-LB163M | VHF | 0.87% | 2.65% | 6.97% | No |
| LB076U-LB078U | VHF | 0.00% | 0.00% | 0.00% | No |
| LB106M-LB119F | VHF | 0.02% | 0.19% | 1.47% | No |
| LB106M-LB122M | VHF | 1.68% | 5.25% | 13.64% | No |
| LB106M-LB124M | VHF | 0.00% | 0.00% | 0.00% | No |
| LB106M-LB129M | VHF | 17.72% | 23.96% | 31.49% | Yes |
| LB106M-LB130F | VHF | 12.92% | 17.56% | 23.29% | Yes |
| LB106M-LB134F | VHF | 0.00% | 0.00% | 0.00% | No |
| LB106M-LB140F | VHF | 0.00% | 0.01% | 0.13% | No |
| LB106M-LB144F | VHF | 0.00% | 0.04% | 0.22% | No |
| LB106M-LB146M | VHF | 0.00% | 0.00% | 0.00% | No |
| LB106M-LB148M | VHF | 0.00% | 0.00% | 0.00% | No |
| LB106M-LB152M | VHF | 34.94% | 43.16% | 52.08% | Yes |
| LB106M-LB154M | VHF | 0.00% | 0.00% | 0.02% | No |
| LB106M-LB157F | VHF | 4.09% | 6.48% | 9.92% | No |
| LB106M-LB163M | VHF | 1.36% | 4.77% | 13.51% | No |
| LB119F-LB122M | VHF | 19.25% | 32.27% | 49.15% | Yes |
| LB119F-LB124M | VHF | 0.00% | 0.00% | 0.00% | No |
| LB119F-LB129M | VHF | 0.00% | 0.00% | 0.00% | No |
| LB119F-LB130F | VHF | 0.00% | 0.00% | 0.00% | No |
| LB119F-LB134F | VHF | 0.00% | 0.00% | 0.00% | No |
| LB119F-LB140F | VHF | 0.00% | 0.00% | 0.00% | No |
| LB119F-LB144F | VHF | 0.00% | 0.00% | 0.00% | No |
| LB119F-LB146M | VHF | 0.00% | 0.04% | 0.64% | No |
| LB119F-LB148M | VHF | 0.00% | 0.00% | 0.00% | No |
| LB119F-LB152M | VHF | 0.00% | 0.01% | 0.45% | No |
| LB119F-LB154M | VHF | 0.00% | 0.00% | 0.00% | No |
| LB119F-LB157F | VHF | 0.00% | 0.00% | 0.00% | No |
| LB119F-LB163M | VHF | 0.00% | 0.00% | 0.07% | No |
| LB122M-LB124M | VHF | 0.00% | 0.00% | 0.00% | No |
| LB122M-LB129M | VHF | 0.00% | 0.00% | 0.00% | No |
| LB122M-LB130F | VHF | 0.00% | 0.00% | 0.00% | No |
| LB122M-LB134F | VHF | 0.00% | 0.00% | 0.00% | No |
| LB122M-LB140F | VHF | 0.00% | 0.00% | 0.00% | No |
| LB122M-LB144F | VHF | 0.00% | 0.00% | 0.00% | No |
| LB122M-LB146M | VHF | 0.00% | 0.01% | 0.11% | No |
| LB122M-LB148M | VHF | 0.00% | 0.00% | 0.00% | No |
| LB122M-LB152M | VHF | 0.00% | 0.00% | 0.00% | No |
| LB122M-LB154M | VHF | 0.00% | 0.00% | 0.00% | No |
| LB122M-LB157F | VHF | 0.00% | 0.00% | 0.00% | No |
| LB122M-LB163M | VHF | 0.00% | 0.00% | 0.00% | No |
| LB124M-LB129M | VHF | 0.00% | 0.00% | 0.00% | No |
| LB124M-LB130F | VHF | 0.00% | 0.00% | 0.00% | No |
| LB124M-LB134F | VHF | 0.00% | 0.00% | 0.00% | No |
| LB124M-LB140F | VHF | 5.94% | 15.29% | 32.54% | Yes |
| LB124M-LB144F | VHF | 0.00% | 0.00% | 0.11% | No |
| LB124M-LB146M | VHF | 2.72% | 8.02% | 19.55% | No |
| LB124M-LB148M | VHF | 0.00% | 0.00% | 0.00% | No |
| LB124M-LB152M | VHF | 0.00% | 0.00% | 0.02% | No |
| LB124M-LB154M | VHF | 0.00% | 0.00% | 0.00% | No |
| LB124M-LB157F | VHF | 0.00% | 0.00% | 0.00% | No |
| LB124M-LB163M | VHF | 0.00% | 0.00% | 0.00% | No |
| LB129M-LB130F | VHF | 16.54% | 23.22% | 31.47% | Yes |
| LB129M-LB134F | VHF | 0.00% | 0.00% | 0.00% | No |
| LB129M-LB140F | VHF | 0.00% | 0.00% | 0.00% | No |
| LB129M-LB144F | VHF | 0.00% | 0.00% | 0.00% | No |
| LB129M-LB146M | VHF | 0.00% | 0.00% | 0.00% | No |
| LB129M-LB148M | VHF | 0.00% | 0.00% | 0.00% | No |
| LB129M-LB152M | VHF | 57.92% | 66.53% | 74.90% | Yes |
| LB129M-LB154M | VHF | 0.00% | 0.00% | 0.00% | No |
| LB129M-LB157F | VHF | 67.82% | 77.08% | 85.41% | Yes |
| LB129M-LB163M | VHF | 1.33% | 4.51% | 12.50% | No |
| LB130F-LB134F | VHF | 0.00% | 0.00% | 0.00% | No |
| LB130F-LB140F | VHF | 0.00% | 0.00% | 0.00% | No |
| LB130F-LB144F | VHF | 0.00% | 0.00% | 0.00% | No |
| LB130F-LB146M | VHF | 0.00% | 0.00% | 0.00% | No |
| LB130F-LB148M | VHF | 0.00% | 0.00% | 0.00% | No |
| LB130F-LB152M | VHF | 3.51% | 11.46% | 29.01% | No |
| LB130F-LB154M | VHF | 0.00% | 0.00% | 0.00% | No |
| LB130F-LB157F | VHF | 0.12% | 0.68% | 2.90% | No |
| LB130F-LB163M | VHF | 0.00% | 0.00% | 0.00% | No |
| LB134F-LB140F | VHF | 0.00% | 0.00% | 0.00% | No |
| LB134F-LB144F | VHF | 0.00% | 0.00% | 0.00% | No |
| LB134F-LB146M | VHF | 0.00% | 0.00% | 0.00% | No |
| LB134F-LB148M | VHF | 0.00% | 0.00% | 0.00% | No |
| LB134F-LB152M | VHF | 0.00% | 0.00% | 0.00% | No |
| LB134F-LB154M | VHF | 0.00% | 0.00% | 0.00% | No |
| LB134F-LB157F | VHF | 0.00% | 0.00% | 0.00% | No |
| LB134F-LB163M | VHF | 0.00% | 0.00% | 0.00% | No |
| LB140F-LB144F | VHF | 6.47% | 13.82% | 26.14% | Yes |
| LB140F-LB146M | VHF | 20.22% | 29.72% | 41.45% | Yes |
| LB140F-LB148M | VHF | 0.00% | 0.00% | 0.00% | No |
| LB140F-LB152M | VHF | 0.00% | 0.00% | 0.05% | No |
| LB140F-LB154M | VHF | 0.23% | 0.96% | 3.34% | No |
| LB140F-LB157F | VHF | 0.00% | 0.00% | 0.00% | No |
| LB140F-LB163M | VHF | 0.00% | 0.00% | 0.00% | No |
| LB144F-LB146M | VHF | 0.07% | 0.58% | 3.27% | No |
| LB144F-LB148M | VHF | 0.00% | 0.00% | 0.00% | No |
| LB144F-LB152M | VHF | 0.00% | 0.01% | 0.12% | No |
| LB144F-LB154M | VHF | 13.86% | 23.55% | 36.86% | Yes |
| LB144F-LB157F | VHF | 0.00% | 0.00% | 0.00% | No |
| LB144F-LB163M | VHF | 0.00% | 0.00% | 0.00% | No |
| LB146M-LB148M | VHF | 0.01% | 0.18% | 1.56% | No |
| LB146M-LB152M | VHF | 0.00% | 0.00% | 0.02% | No |
| LB146M-LB154M | VHF | 0.00% | 0.00% | 0.00% | No |
| LB146M-LB157F | VHF | 0.00% | 0.00% | 0.00% | No |
| LB146M-LB163M | VHF | 0.00% | 0.00% | 0.00% | No |
| LB148M-LB152M | VHF | 0.00% | 0.00% | 0.00% | No |
| LB148M-LB154M | VHF | 0.00% | 0.01% | 0.16% | No |
| LB148M-LB157F | VHF | 0.00% | 0.00% | 0.00% | No |
| LB148M-LB163M | VHF | 0.00% | 0.00% | 0.00% | No |
| LB152M-LB154M | VHF | 0.00% | 0.00% | 0.00% | No |
| LB152M-LB157F | VHF | 50.94% | 60.38% | 69.84% | Yes |
| LB152M-LB163M | VHF | 0.00% | 0.02% | 0.12% | No |
| LB154M-LB157F | VHF | 0.00% | 0.00% | 0.00% | No |
| LB154M-LB163M | VHF | 0.00% | 0.00% | 0.00% | No |
| LB157F-LB163M | VHF | 0.00% | 0.00% | 0.01% | No |
| SAB04F-SAB05F | VHF | 0.00% | 0.01% | 0.09% | No |
| SAB04F-SAB06M | VHF | 0.00% | 0.00% | 0.00% | No |
| SAB04F-SAB07M | VHF | 0.00% | 0.00% | 0.00% | No |
| SAB04F-SAB08M | VHF | 0.00% | 0.00% | 0.00% | No |
| SAB04F-SAB10F | VHF | 0.00% | 0.00% | 0.00% | No |
| SAB04F-SAB13M | VHF | 0.00% | 0.00% | 0.00% | No |
| SAB04F-SAB15M | VHF | 0.00% | 0.00% | 0.00% | No |
| SAB04F-SAB19F | VHF | 0.00% | 0.00% | 0.00% | No |
| SAB05F-SAB06M | VHF | 78.22% | 85.50% | 91.62% | Yes |
| SAB05F-SAB07M | VHF | 93.89% | 98.22% | 99.94% | Yes |
| SAB05F-SAB08M | VHF | 4.02% | 7.47% | 13.00% | No |
| SAB05F-SAB10F | VHF | 0.01% | 0.04% | 0.22% | No |
| SAB05F-SAB13M | VHF | 0.00% | 0.00% | 0.00% | No |
| SAB05F-SAB15M | VHF | 0.00% | 0.00% | 0.00% | No |
| SAB05F-SAB19F | VHF | 0.65% | 1.32% | 2.54% | No |
| SAB06M-SAB07M | VHF | 84.61% | 91.85% | 97.00% | Yes |
| SAB06M-SAB08M | VHF | 9.56% | 17.65% | 29.72% | Yes |
| SAB06M-SAB10F | VHF | 0.00% | 0.00% | 0.00% | No |
| SAB06M-SAB13M | VHF | 0.00% | 0.00% | 0.00% | No |
| SAB06M-SAB15M | VHF | 1.07% | 5.18% | 17.95% | No |
| SAB06M-SAB19F | VHF | 5.49% | 12.91% | 26.21% | No |
| SAB07M-SAB08M | VHF | 4.28% | 8.52% | 15.61% | No |
| SAB07M-SAB10F | VHF | 0.00% | 0.01% | 0.05% | No |
| SAB07M-SAB13M | VHF | 0.00% | 0.00% | 0.00% | No |
| SAB07M-SAB15M | VHF | 0.00% | 0.01% | 0.04% | No |
| SAB07M-SAB19F | VHF | 0.64% | 1.51% | 3.29% | No |
| SAB08M-SAB10F | VHF | 1.30% | 2.33% | 3.99% | No |
| SAB08M-SAB13M | VHF | 0.00% | 0.00% | 0.00% | No |
| SAB08M-SAB15M | VHF | 54.12% | 63.15% | 72.08% | Yes |
| SAB08M-SAB19F | VHF | 50.36% | 57.03% | 63.79% | Yes |
| SAB10F-SAB13M | VHF | 0.00% | 0.00% | 0.00% | No |
| SAB10F-SAB15M | VHF | 0.00% | 0.00% | 0.00% | No |
| SAB10F-SAB19F | VHF | 0.01% | 0.09% | 0.42% | No |
| SAB13M-SAB15M | VHF | 0.00% | 0.00% | 0.00% | No |
| SAB13M-SAB19F | VHF | 0.00% | 0.00% | 0.00% | No |
| SAB15M-SAB19F | VHF | 9.60% | 14.43% | 20.87% | Yes |
| WB07F-WB12M | VHF | 59.62% | 62.06% | 64.50% | Yes |
| WB07F-WB32F | VHF | 23.32% | 25.77% | 28.37% | Yes |
| WB07F-WB39F | VHF | 14.01% | 15.84% | 17.83% | Yes |
| WB07F-WB40F | VHF | 0.01% | 0.01% | 0.02% | No |
| WB07F-WB42M | VHF | 21.09% | 24.38% | 27.99% | Yes |
| WB07F-WB44M | VHF | 6.50% | 11.55% | 19.18% | No |
| WB12M-WB32F | VHF | 50.48% | 53.38% | 56.33% | Yes |
| WB12M-WB39F | VHF | 62.10% | 65.81% | 69.50% | Yes |
| WB12M-WB40F | VHF | 12.27% | 16.52% | 21.75% | Yes |
| WB12M-WB42M | VHF | 50.03% | 53.75% | 57.52% | Yes |
| WB12M-WB44M | VHF | 18.63% | 27.53% | 38.66% | Yes |
| WB32F-WB39F | VHF | 16.67% | 21.78% | 27.86% | Yes |
| WB32F-WB40F | VHF | 0.00% | 0.01% | 0.02% | No |
| WB32F-WB42M | VHF | 93.39% | 95.92% | 97.87% | Yes |
| WB32F-WB44M | VHF | 0.17% | 0.69% | 2.43% | No |
| WB39F-WB40F | VHF | 29.79% | 36.45% | 43.79% | Yes |
| WB39F-WB42M | VHF | 15.30% | 20.53% | 26.88% | Yes |
| WB39F-WB44M | VHF | 24.75% | 33.47% | 43.66% | Yes |
| WB40F-WB42M | VHF | 0.00% | 0.00% | 0.01% | No |
| WB40F-WB44M | VHF | 80.10% | 88.02% | 94.25% | Yes |
| WB42M-WB44M | VHF | 0.00% | 0.01% | 0.06% | No |
| WB47M-WB48F | VHF | 3.56% | 5.70% | 8.79% | No |
| WB48F-WB55M | VHF | 0.01% | 0.05% | 0.22% | No |
| WB48F-WB53M | VHF | 1.21% | 2.91% | 6.34% | No |
| WB53M-WB55M | VHF | 0.00% | 0.01% | 0.15% | No |
| EB22F-EB23F | GPS | 0.00% | 0.00% | 0.00% | No |
| EB22F-EB24M | GPS | 0.00% | 0.00% | 0.00% | No |
| EB23F-EB24M | GPS | 0.08% | 0.16% | 0.33% | No |
| EB25M-EB26F | GPS | 92.50% | 94.45% | 96.12% | Yes |
| EB25M-EB27M | GPS | 0.43% | 0.70% | 1.13% | No |
| EB25M-EB28F | GPS | 3.69% | 4.80% | 6.17% | No |
| EB25M-EB29F | GPS | 0.17% | 0.35% | 0.69% | No |
| EB25M-EB30F | GPS | 8.44% | 10.85% | 13.77% | No |
| EB26F-EB27M | GPS | 0.19% | 0.35% | 0.63% | No |
| EB26F-EB28F | GPS | 2.00% | 2.83% | 3.92% | No |
| EB26F-EB29F | GPS | 0.06% | 0.14% | 0.32% | No |
| EB26F-EB30F | GPS | 5.61% | 7.60% | 10.14% | No |
| EB27M-EB28F | GPS | 0.01% | 0.02% | 0.03% | No |
| EB27M-EB29F | GPS | 73.77% | 79.40% | 84.60% | Yes |
| EB27M-EB30F | GPS | 13.01% | 15.70% | 18.78% | Yes |
| EB28F-EB29F | GPS | 0.01% | 0.01% | 0.02% | No |
| EB28F-EB30F | GPS | 7.34% | 8.90% | 10.71% | No |
| EB29F-EB30F | GPS | 10.76% | 14.58% | 19.33% | Yes |
| EB35M-EB36M | GPS | 4.08% | 11.14% | 25.23% | No |
| EB35M-EB37M | GPS | 0.81% | 1.91% | 4.16% | No |
| EB35M-EB40F | GPS | 0.63% | 1.48% | 3.21% | No |
| EB36M-EB37M | GPS | 5.77% | 7.93% | 10.70% | No |
| EB36M-EB40F | GPS | 8.31% | 11.37% | 15.23% | No |
| EB37M-EB40F | GPS | 76.61% | 83.00% | 88.66% | Yes |
| EB63F-EB64F | GPS | 0.00% | 0.00% | 0.00% | No |
| EB63F-EB65M | GPS | 0.02% | 0.38% | 3.99% | No |
| EB63F-EB77M | GPS | 32.08% | 37.21% | 42.73% | Yes |
| EB63F-EB78M | GPS | 0.00% | 0.00% | 0.00% | No |
| EB63F-EB79M | GPS | 0.00% | 0.00% | 0.00% | No |
| EB63F-EB64F | GPS | 0.00% | 0.00% | 0.00% | No |
| EB63F-EB67M | GPS | 19.17% | 24.57% | 30.88% | Yes |
| EB63F-EB68M | GPS | 0.05% | 0.06% | 0.08% | No |
| EB63F-EB69F | GPS | 0.00% | 0.00% | 0.00% | No |
| EB63F-EB70M | GPS | 0.00% | 0.00% | 0.03% | No |
| EB63F-EB72M | GPS | 13.57% | 18.44% | 24.43% | Yes |
| EB63F-EB81F | GPS | 0.00% | 0.00% | 0.00% | No |
| EB63F-EB83F | GPS | 0.00% | 0.00% | 0.16% | No |
| EB63F-EB80M | GPS | 0.00% | 0.00% | 0.00% | No |
| EB64F-EB65M | GPS | 35.31% | 53.53% | 73.05% | Yes |
| EB64F-EB67M | GPS | 23.22% | 32.91% | 44.51% | Yes |
| EB64F-EB68M | GPS | 0.01% | 0.02% | 0.04% | No |
| EB64F-EB69F | GPS | 0.00% | 0.00% | 0.00% | No |
| EB64F-EB70M | GPS | 0.00% | 0.00% | 0.00% | No |
| EB64F-EB72M | GPS | 21.29% | 27.52% | 34.77% | Yes |
| EB64F-EB80M | GPS | 0.00% | 0.00% | 0.00% | No |
| EB64F-EB77M | GPS | 0.00% | 0.00% | 0.00% | No |
| EB64F-EB78M | GPS | 0.00% | 0.00% | 0.00% | No |
| EB64F-EB79M | GPS | 0.00% | 0.00% | 0.00% | No |
| EB64F-EB81F | GPS | 0.00% | 0.00% | 0.00% | No |
| EB64F-EB83F | GPS | 0.00% | 0.00% | 0.02% | No |
| EB67M-EB68M | GPS | 25.98% | 34.54% | 44.41% | Yes |
| EB67M-EB69F | GPS | 17.97% | 23.98% | 31.18% | Yes |
| EB67M-EB70M | GPS | 22.09% | 39.71% | 61.89% | Yes |
| EB67M-EB72M | GPS | 62.89% | 79.75% | 92.92% | Yes |
| EB67M-EB77M | GPS | 28.90% | 37.58% | 47.39% | Yes |
| EB67M-EB78M | GPS | 1.47% | 7.18% | 24.19% | No |
| EB67M-EB79M | GPS | 0.57% | 3.89% | 16.97% | No |
| EB67M-EB80M | GPS | 0.13% | 1.04% | 5.73% | No |
| EB67M-EB81F | GPS | 0.03% | 0.33% | 2.43% | No |
| EB67M-EB83F | GPS | 0.41% | 3.20% | 15.44% | No |
| EB68M-EB69F | GPS | 71.84% | 75.30% | 78.65% | Yes |
| EB68M-EB70M | GPS | 1.76% | 3.02% | 4.98% | No |
| EB68M-EB72M | GPS | 7.79% | 13.46% | 21.79% | No |
| EB68M-EB77M | GPS | 29.94% | 35.49% | 41.53% | Yes |
| EB68M-EB78M | GPS | 0.00% | 0.00% | 0.00% | No |
| EB68M-EB79M | GPS | 0.00% | 0.00% | 0.00% | No |
| EB68M-EB80M | GPS | 0.00% | 0.00% | 0.00% | No |
| EB68M-EB81F | GPS | 0.00% | 0.00% | 0.00% | No |
| EB68M-EB83F | GPS | 0.00% | 0.06% | 1.63% | No |
| EB69F-EB70M | GPS | 1.87% | 4.06% | 8.10% | No |
| EB69F-EB72M | GPS | 6.21% | 10.90% | 17.95% | No |
| EB69F-EB77M | GPS | 12.60% | 16.74% | 21.79% | Yes |
| EB69F-EB78M | GPS | 0.00% | 0.00% | 0.00% | No |
| EB69F-EB79M | GPS | 0.00% | 0.00% | 0.00% | No |
| EB69F-EB80M | GPS | 0.00% | 0.00% | 0.00% | No |
| EB69F-EB81F | GPS | 0.00% | 0.00% | 0.00% | No |
| EB69F-EB83F | GPS | 0.00% | 0.03% | 1.25% | No |
| EB70M-EB72M | GPS | 0.71% | 2.25% | 6.11% | No |
| EB70M-EB77M | GPS | 0.29% | 0.94% | 2.65% | No |
| EB70M-EB78M | GPS | 0.23% | 0.83% | 2.60% | No |
| EB70M-EB79M | GPS | 0.00% | 0.00% | 0.01% | No |
| EB70M-EB80M | GPS | 0.00% | 0.00% | 0.00% | No |
| EB70M-EB81F | GPS | 0.00% | 0.00% | 0.04% | No |
| EB70M-EB83F | GPS | 0.06% | 0.72% | 5.25% | No |
| EB72M-EB77M | GPS | 12.34% | 18.74% | 27.17% | Yes |
| EB72M-EB78M | GPS | 0.00% | 0.00% | 0.00% | No |
| EB72M-EB79M | GPS | 0.00% | 0.00% | 0.03% | No |
| EB72M-EB80M | GPS | 0.00% | 0.00% | 0.02% | No |
| EB72M-EB81F | GPS | 0.00% | 0.00% | 0.02% | No |
| EB72M-EB83F | GPS | 0.00% | 0.00% | 0.00% | No |
| EB77M-EB78M | GPS | 0.00% | 0.00% | 0.00% | No |
| EB77M-EB79M | GPS | 0.00% | 0.00% | 0.00% | No |
| EB77M-EB80M | GPS | 0.00% | 0.00% | 0.00% | No |
| EB77M-EB81F | GPS | 0.00% | 0.00% | 0.00% | No |
| EB77M-EB83F | GPS | 0.00% | 0.01% | 0.49% | No |
| EB78M-EB79M | GPS | 2.64% | 4.08% | 6.14% | No |
| EB78M-EB80M | GPS | 61.26% | 72.29% | 82.48% | Yes |
| EB78M-EB81F | GPS | 10.71% | 18.41% | 29.38% | Yes |
| EB78M-EB83F | GPS | 13.17% | 24.67% | 41.19% | Yes |
| EB79M-EB80M | GPS | 2.96% | 4.61% | 6.97% | No |
| EB79M-EB81F | GPS | 0.19% | 0.51% | 1.23% | No |
| EB79M-EB83F | GPS | 22.07% | 37.77% | 57.50% | Yes |
| EB80M-EB81F | GPS | 11.24% | 17.63% | 26.28% | Yes |
| EB80M-EB83F | GPS | 13.34% | 22.54% | 35.23% | Yes |
| EB81F-EB83F | GPS | 37.70% | 61.02% | 83.94% | Yes |
| HB727054F-HB729230M | GPS | 77.42% | 86.91% | 94.26% | Yes |
| HB727054F-HB729233M | GPS | 26.17% | 31.79% | 38.03% | Yes |
| HB727054F-HB729234F | GPS | 4.20% | 5.39% | 6.84% | No |
| HB729230M-HB729233M | GPS | 39.18% | 51.72% | 65.05% | Yes |
| HB729230M-HB729234F | GPS | 16.57% | 24.62% | 34.84% | Yes |
| HB729233M-HB729234F | GPS | 30.26% | 35.92% | 42.09% | Yes |
| HR02F-HR03M | GPS | 13.13% | 15.96% | 19.20% | Yes |
| HR02F-HR04F | GPS | 4.00% | 6.12% | 9.10% | No |
| HR02F-HR05F | GPS | 0.01% | 0.11% | 1.07% | No |
| HR02F-HR06M | GPS | 0.20% | 0.50% | 1.19% | No |
| HR02F-HR07F | GPS | 0.04% | 0.18% | 0.67% | No |
| HR02F-HR09F | GPS | 0.22% | 0.81% | 2.57% | No |
| HR03M-HR04F | GPS | 67.85% | 76.08% | 83.63% | Yes |
| HR03M-HR05F | GPS | 0.00% | 0.00% | 0.00% | No |
| HR03M-HR06M | GPS | 1.08% | 1.87% | 3.14% | No |
| HR03M-HR07F | GPS | 0.11% | 0.33% | 0.93% | No |
| HR03M-HR09F | GPS | 0.45% | 1.57% | 4.60% | No |
| HR04F-HR05F | GPS | 0.00% | 0.00% | 0.00% | No |
| HR04F-HR06M | GPS | 0.00% | 0.00% | 0.00% | No |
| HR04F-HR07F | GPS | 0.00% | 0.00% | 0.00% | No |
| HR04F-HR09F | GPS | 0.06% | 0.36% | 1.78% | No |
| HR05F-HR06M | GPS | 55.67% | 62.69% | 69.67% | Yes |
| HR05F-HR07F | GPS | 60.40% | 68.95% | 77.15% | Yes |
| HR05F-HR09F | GPS | 31.64% | 37.26% | 43.34% | Yes |
| HR06M-HR07F | GPS | 34.84% | 38.82% | 43.01% | Yes |
| HR06M-HR09F | GPS | 16.10% | 18.70% | 21.59% | Yes |
| HR07F-HR09F | GPS | 14.32% | 16.08% | 17.99% | Yes |
| KRB2M-KRB3F | GPS | 75.04% | 81.21% | 86.79% | Yes |
| KRB2M-KRB4F | GPS | 0.00% | 0.00% | 0.01% | No |
| KRB3F-KRB4F | GPS | 0.00% | 0.00% | 0.00% | No |
| KRB5F-KRB6M | GPS | 5.25% | 7.24% | 9.81% | No |
| KRB5F-KRB8M | GPS | 1.03% | 2.82% | 6.85% | No |
| KRB5F-KRB9M | GPS | 85.83% | 89.31% | 92.39% | Yes |
| KRB6M-KRB8M | GPS | 2.04% | 5.45% | 12.65% | No |
| KRB6M-KRB9M | GPS | 6.72% | 9.67% | 13.57% | No |
| KRB8M-KRB9M | GPS | 0.52% | 1.59% | 4.31% | No |
| LB726981M-LB727047M | GPS | 8.16% | 14.64% | 24.34% | Yes |
| LB726981M-LB727051F | GPS | 0.00% | 0.06% | 0.61% | No |
| LB726981M-LB727052M | GPS | 0.00% | 0.00% | 0.00% | No |
| LB727047M-LB727051F | GPS | 0.00% | 0.00% | 0.00% | No |
| LB727047M-LB727052M | GPS | 0.00% | 0.00% | 0.00% | No |
| LB727051F-LB727052M | GPS | 0.00% | 0.00% | 0.00% | No |

*High overlap were those individuals who had percent home range overlap greater than the mean home range overlap of 13.2%.

Literature Cited

Beltrán JF, Tewes ME.1995. Immobilization of ocelots and bobcats with ketamine hydrochloride and xylazine hydrochloride. Journal of Wildlife Diseases 31(1):43–48. <https://doi.org/10.7589/0090-3558-31.1.43>

Blankenship TL. 2000. Ecological response of bobcats to fluctuating prey populations on the Welder Wildlife Foundation Refuge. Dissertation, Texas A&M University-Kingsville.

Blankenship TL, Haines AM, Tewes ME, Silvy NJ. 2006. Comparing survival and cause-specific mortality between resident and transient bobcats *Lynx rufus*. Wildlife Biology 12:297–303. [http://dx.doi.org/10.2981/0909-6396(2006)12[297:CSACMB]2.0.CO;2](http://dx.doi.org/10.2981/0909-6396(2006)12%5b297:CSACMB%5d2.0.CO;2)

Branney AB, Veals Dutt AM, Wardle ZM, Tanner EP, Tewes ME, Cherry MJ. 2024*.* Scale of effect of landscape patterns on bobcat (*Lynx rufus*) resource selection in a multi-use rangeland. Landscape Ecology 39:147. <https://doi.org/10.1007/s10980-024-01944-7>

Fischer CV. Habitat use by free-ranging felids in an agroecosystem. Thesis. Texas A&M University-Kingsville

Harveson PM, Tewes ME, Anderson GL, Laack LL. Habitat use by ocelots in south Texas: implications for habitat restoration. Wildlife Society Bulletin 32(3):948–954. [https://doi.org/10.2193/0091-7648(2004)032[0948:HUBOIS]2.0.CO;2](https://doi.org/10.2193/0091-7648(2004)032%5b0948:HUBOIS%5d2.0.CO;2)

Korn JM. 2013. Genetic pedigree of ocelot and fine scale movement patterns of bobcat in South Texas. Dissertation. Texas A&M University-Kingsville.

Laack LL. 1991. Ecology of ocelot in South Texas. Thesis. Texas A&I University.

Leonard JP, Tewes ME, Lombardi JV, Wester DW, Campbell TA. 2020. Effects of sun angle, lunar illumination, and diurnal temperature on temporal movement rates of sympatric ocelots and bobcats in South Texas. PLOS ONE 15(4):e0231732. <https://doi.org/10.1371/journal.pone.0231732>

Rockhill AP, Chinnadurai SK, Powell RA, DePerno CS. 2011. A comparison of two field chemical immobilizations techniques for bobcats (*Lynx rufus*). Journal of Zoo and Wildlife Medicine 42(4):580–584. <https://www.jstor.org/stable/41417161>

Sergeyev M, Holbrook JD, Lombardi JV, Tewes ME, Campbell TA. 2023. Behaviorally mediated coexistence of ocelots, bobcats and coyotes using hidden Markov models. Oikos 2023(4):e09480. <https://doi.org/10.1111/oik.09480>
